# Oleic acid restores the impaired antitumor immunity of γδ-T cells induced by palmitic acid

**DOI:** 10.1038/s41392-025-02295-8

**Published:** 2025-07-03

**Authors:** Yanmei Zhang, Zheng Xiang, Yan Xu, Lo Sha Cheung, Xiwei Wang, Manni Wang, Howard Ho Wai Wong, Zhenyao Zhu, Wenyue Zhang, Yifan Gao, Xianze Luo, Yin Celeste Cheuk, Yixin Zhou, Xianfeng Zha, Yashi Chen, Man Li, Feifei Luo, Yiwei Chu, Yu-Lung Lau, Yinping Liu, Wenwei Tu

**Affiliations:** 1 Department of Paediatrics & Adolescent Medicine, Li Ka Shing Faculty of Medicine, The University of Hong Kong, Hong Kong, China; 2 Department of Microbiology and Immunology, Health Science Center, School of Medicine, Jinan University, Guangzhou, Guangdong China; 3 Key Laboratory of Viral Pathogenesis & Infection Prevention and Control, Jinan University, Ministry of Education, Guangzhou, Guangdong China; 4 Guangdong Provincial Key Laboratory of Tumor Interventional Diagnosis and Treatment, Zhuhai Institute of Translational Medicine, Zhuhai People’s Hospital (Zhuhai Clinical Medical College of Jinan University), Jinan University, Zhuhai, Guangdong China; 5 State Key Laboratory of Bioactive Molecules and Druggability Assessment, The Biomedical Translational Research Institute, Health Science Center, School of Medicine, Jinan University, Guangzhou, Guangdong China; 6 Department of Clinical Laboratory, The First Affiliated Hospital, Jinan University, Guangzhou, Guangdong China; 7 Department of Digestive Diseases, Huashan Hospital, Fudan University, Shanghai, China; 8 Department of Immunology, School of Basic Medical Sciences, Biotherapy Research Center and Institutes of Biomedical Sciences, Fudan University, Shanghai, China; 9 CAS Key Laboratory of Quantitative Engineering Biology, Shenzhen Institute of Synthetic Biology, Shenzhen Institute of Advanced Technology, Chinese Academy of Sciences, Shenzhen, China

**Keywords:** Tumour immunology, Cancer therapy

## Abstract

Dietary fatty acids (FAs) are associated with the therapeutic intervention under various health conditions. Human γδ-T cells are indispensable for immunosurveillance toward malignant cells. However, their impact on γδ-T cell metabolism and function remains poorly unexplored. Here, we applied targeted metabolomics analysis to serum FAs among cancer patients undergoing γδ-T cell therapy and discovered that palmitic acid (PA) or oleic acid (OA) levels were associated with the efficacy of Vγ9Vδ2-T cell therapy. We further elucidated that PA suppresses the antitumor activity of Vγ9Vδ2-T cells by disrupting metabolic processes and inhibiting the secretion of lytic granules, whereas OA restores the impaired antitumor activity of Vγ9Vδ2-T cells. Mechanistically, we surprisingly found that PA stimulates Vγ9Vδ2-T cells to secrete excessive IFNγ, which in turn induces cell pyroptosis, ultimately resulting in decreased antitumor activity. In contrast, OA reduces IFNγ secretion and mitigates cell pyroptosis, thereby restoring their antitumor activity. Alternatively, direct blockade of IFNγ by anti-IFNγ mAb or inhibition of pyroptosis by dimethyl fumarate (DMF) also restores their antitumor activity. This study highlights a novel mechanism whereby dietary FAs modulate γδ-T cell function through regulating IFNγ-mediated pyroptosis. Additionally, it offers proof-of-concept for an innovative approach by targeting IFNγ-mediated pyroptosis or dietary OA supplementation to strengthen the antitumor immunity of γδ-T cells against cancers.

## Introduction

The nutritional composition of our diets profoundly influences human physiology, contributing to both adaptive benefits and potential detriments.^1^ Among essential nutrients, fatty acids (FAs) are indispensable for normal human growth and development and have been implicated in the prevention of various tumors. However, studying the effects of FAs on cancer progression remains challenging due to limitations such as the difficulty of controlling dietary intake in clinical patients and the lack of robust comparative studies for different FAs. Notably, recent research has highlighted the immunomodulatory properties of FAs, particularly their utilization by immune cells to regulate cancer immunity. For example, linoleic acid has been shown to alter mitochondrial function, which enhances the cytotoxic T cell memory phenotype, consequently boosting the efficacy of chimeric antigen receptor (CAR)-T cell therapy against tumors.^2^ Elaidic acid enhances the presentation of tumor antigens, and its supplementation in diets has been associated with improved cancer immunotherapy outcomes.^3^ Moreover, dietary trans-vaccenic acid (TVA) can promote the infiltration and cytotoxic functions of effector CD8^+^ T cells within cancers, subsequently enhancing antitumor immunity.^4^ While these studies underscore the diverse roles of individual FAs in modulating immune responses, the effects of specific FAs on tumor progression and antitumor immunity vary significantly. Despite these advances, a critical gap remains in understanding how different dietary FAs directly influence T-cell-mediated antitumor activity. Addressing this gap is crucial to fully uncover the potential of FAs in cancer immunotherapy and for developing targeted dietary strategies to optimize immune responses against tumors.

As a unique cluster of T cells, γδ-T cells possess characteristics of αβ-T cells, antigen-presenting cells (APCs), and natural killer (NK) cells. They mediate MHC-unrestricted cytotoxicity against virus-infected and tumor cells through granzyme/perforin release and cytokine production.^5–10^ Notably, γδ-T cell signatures in the tumor were identified as the most favorable markers for prognosis among 22 immune cell populations for cancers.^11^ Our previous reports proved that pamidronate (PAM)-activated Vγ9Vδ2-T cells and their exosomes efficiently suppress EBV-associated tumor growth.^10,12–14^ Furthermore, adoptive allogeneic γδ-T cell transfer has demonstrated improved lifespan and encouraging clinical safety in late-stage liver and lung cancer patients.^15^ These findings underscore the pivotal role of γδ-T cells in immunosurveillance and their potential as promising candidates for immunotherapy.

However, clinical applications of Vγ9Vδ2-T cell therapy have demonstrated promising yet variable outcomes.^16–18^ A pilot study in four patients with hematological malignancies demonstrated that adoptive Vγ9Vδ2-T cell transfer, combined with zoledronate and IL-2, induced complete remission in three patients, sustained for over 6 months.^16^ A phase I evaluation reported stable disease in 5 of 10 metastatic breast cancer patients by using expanded Vγ9Vδ2-T cells, though no complete responses were observed.^17^ In non-small cell lung cancer (NSCLC), a phase I study with allogeneic Vγ9Vδ2-T cells achieved partial responses in 16% of patients, with a 12-month median overall survival in responders.^18^ The variability in outcomes associated with Vγ9Vδ2-T cell therapy might be ascribed to several factors, encompassing the limited patient sample sizes, advanced disease stages, prior treatments, tumor heterogeneity, and individual patient characteristics. Furthermore, tumor microenvironment (TME) can hinder Vγ9Vδ2-T cell functionality through immunosuppressive cells and metabolic stressors. Challenges also encompass refining ex vivo expansion techniques, ensuring consistent activation, and overcoming TME-induced resistance. Consequently, ongoing investigations are exploring strategies to booster effector function, with a focus on targeting metabolic vulnerabilities, in a bid to enhance clinical outcomes. So far, the influence of specific dietary FAs in the Vγ9Vδ2-T cell activation, expansion, and antitumor properties remains largely unexplored.

Here, we employed targeted metabolomics to measure FA levels in the serum of cancer patients undergoing γδ-T cell therapy. Our analysis revealed correlations between serum levels of PA or OA and the efficacy of γδ-T cell therapy. Subsequently, we examined the impact of PA or OA on the metabolic processes, protein expression, and functions of Vγ9Vδ2-T cells, and their contributions to tumor development. Our findings provide compelling evidence that specific dietary FAs influence the functional characteristics of Vγ9Vδ2-T cells, shedding light on their potential contributions to variable cancer and infection risks among individuals. These results support the notion that dietary supplementation of specific FAs could regulate innate immunosurveillance of γδ-T cells against cancers via modulating the antitumor function.

## Results

### The level of PA or OA is associated with the efficacy of Vγ9Vδ2-T cell-based anticancer therapy in patients

**Fig. 1:**
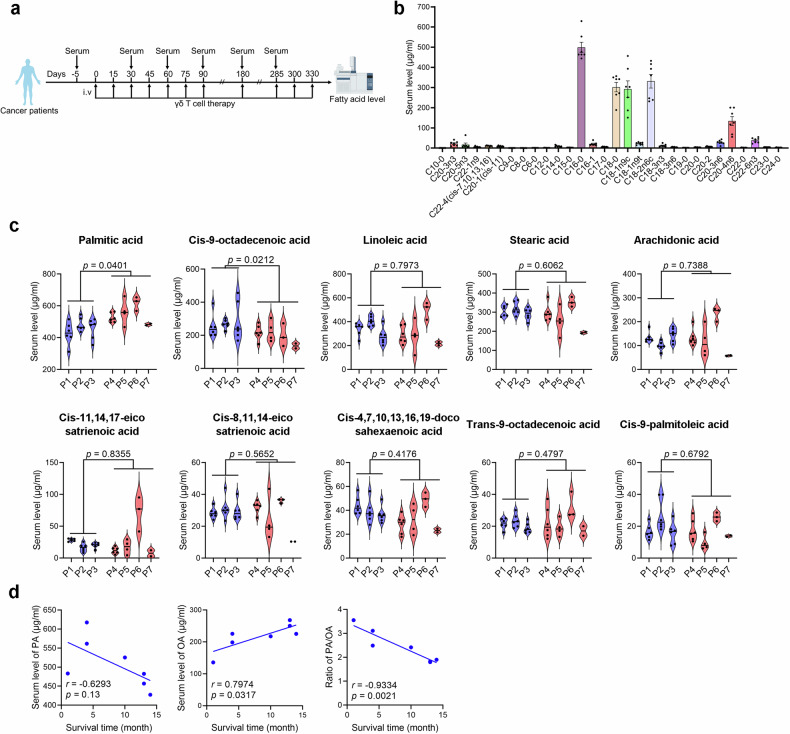
The level of PA or OA is associated with the efficacy of Vγ9Vδ2-T cell-based anticancer therapy in patients. **a** Diagram showing the protocol for collecting serum of HCC patients treated with Vγ9Vδ2-T cells. **b** Mass spectrometric analysis of FAs in serum of cancer patients before Vγ9Vδ2-T cell therapy (*n* = 7). **c** Serum FFA levels of seven cancer patients who received Vγ9Vδ2-T cell treatment are displayed in violin plots. FA levels were measured at baseline and before each Vγ9Vδ2-T cell infusion, corresponding to all timepoints in (**a**), for responders and non-responders until therapy cessation. Patients with complete response to Vγ9Vδ2-T cell therapy are represented by blue violin plots, and patients with progressive disease under Vγ9Vδ2-T cell therapy are represented by red violin plots. **d** Correlation between average serum level of PA, OA, or ratio of PA/OA and survival time of patients. The data are shown as the mean ± SEM

To determine whether FAs are associated with the efficacy of Vγ9Vδ2-T cell-based anticancer therapy, we collected the serum from seven patients with liver cancer under Vγ9Vδ2-T cell therapy and performed targeted metabolomics to detect serum levels of FAs (Fig. 1a). Three patients were recognized as responders because they had complete response to Vγ9Vδ2-T cell therapy with improved progression-free survival, while four patients were non-responders since they were resistant to Vγ9Vδ2-T cell therapy and died during the therapeutic course. Our analysis revealed that PA was the most abundant FA in human serum while OA, stearic acid, and linoleic acid had similar levels in serum (Fig. 1b). We further analyzed the top 10 FA levels in serum and found that only the level of PA was significantly lower, and the level of OA was significantly higher in responders than that in non-responders (Fig. 1c). Moreover, we found that although the mean of serum PA showed no significant correlation with the survival time of patients, the mean of serum OA was positively correlated with the survival time of patients. Importantly, the ratio of PA/OA was negatively related to the survival time of patients (Fig. 1d). These results suggested that the level of PA or OA might be associated with the outcome of Vγ9Vδ2-T cell-based anticancer therapy in patients. Moreover, the ratio of PA/OA could potentially act as a marker for predicting the therapeutic effectiveness of Vγ9Vδ2-T cells in cancer patients.

### OA restores the reduced antitumor activity of Vγ9Vδ2-T cells induced by PA

To explore how PA or OA affect the function of Vγ9Vδ2-T cells, we expanded Vγ9Vδ2-T cells from peripheral blood mononuclear cells (PBMCs) of healthy donors by PAM with the addition of PA and/or OA in vitro. As CD36, a scavenger receptor, facilitates the high-affinity uptake of long-chain FAs in tissue,^19^ we initially assessed the expression of CD36 across various immune cells from resting PBMCs. Vγ9Vδ2-T cells showed higher expression of CD36 than other immune cells, including CD4 T cells, CD8 T cells, NK cells, and Vδ1-T cells (Supplementary Fig. 1a), suggesting that Vγ9Vδ2-T cells may be more susceptible to FAs than other immune cells. After PAM stimulation for 5 days, the percentage of CD36-positive Vγ9Vδ2-T cells exhibited a reduction from approximately 60% at baseline (Supplementary Fig. 1a) to less than 40% (Supplementary Fig. 1b), indicating that CD36 expression was downregulated in Vγ9Vδ2-T cells. PA treatment increased CD36 expression while OA treatment decreased CD36 expression in Vγ9Vδ2-T cells, and OA could abolish the increase of CD36 expression induced by PA (Supplementary Fig. 1b). Fatty acid-binding proteins (FABPs) and peroxisome proliferator-activated receptors (PPARs) also have essential functions in FA sensing and transport.^20^ Here, we further examined the expression of FABP4, FABP5, PPARδ, and PPARγ in PAM-expanded human Vγ9Vδ2-T cells treated by PA or OA. The FABP4 and PPARδ were not detectable in Vγ9Vδ2-T cells treated by PA or OA. Vγ9Vδ2-T cells expressed FABP5 and PPARγ, however, their expression levels had no significant changes after PA or OA treatment (Supplementary Fig. 1c). To determine whether PA or OA causes similar lipid accumulation. Microscopy analysis revealed that PA treatment resulted in intracellular phospholipid accumulation, while OA treatment caused neutral lipids accumulation in Vγ9Vδ2-T cells compared to the BSA group (Supplementary Fig. 1d). Additionally, lipidtox analysis also demonstrated that OA-treated Vγ9Vδ2-T cells readily incorporated OA from the media (Supplementary Fig. 1e).

**Fig. 2:**
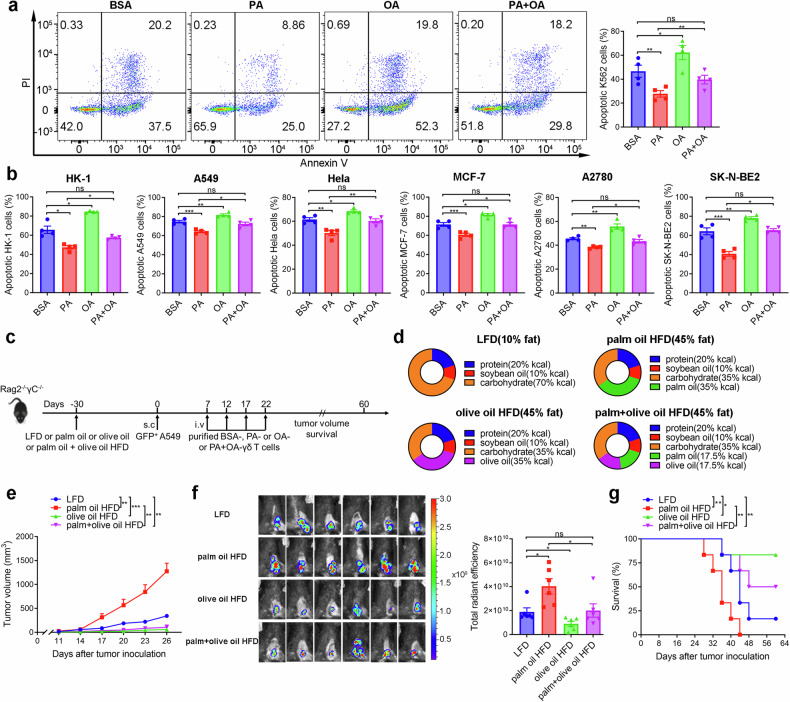
OA restores the reduced antitumor activity of Vγ9Vδ2-T cells induced by PA. **a** The apoptotic K562 cells were determined by flow cytometry after coculture with Vγ9Vδ2-T cells for 6 h (*n* = 4). **b** The percentages of apoptotic tumor cells after coculture with Vγ9Vδ2-T cells for 6 h (*n* = 4). **c** Diagram of the protocol in (**d**–**g**). Rag2^−/−^γc^−/−^ mice fed on the LFD, palm oil, olive oil or palm and olive oil HFDs for 30 days were subcutaneously (s.c.) injected with GFP^+^ A549 tumor cells. Expanded BSA-, PA-, OA- or PA + OA-Vγ9Vδ2-T cells were intravenously (i.v.) transferred into the mice at the indicated time (*n* = 6 mice per group). **d** Main components of control LFD, palm oil, olive oil, and palm and olive oil HFDs. **e** Tumor volumes were obtained at the indicated time. **f** On day 26 following GFP^+^ A549 tumor cells inoculation, whole-body fluorescence images (left) and total radiant efficiency of fluorescence intensity (right) of the mice are shown after Vγ9Vδ2-T cells treatment. **g** Survival curves were acquired at the specified time. Quantitative data are shown as the mean ± SEM. ns not significant; *p < 0.05; **p < 0.01; ***p < 0.001

Next, the capacity of Vγ9Vδ2-T cells to fight cancers under PA or OA treatment was investigated. In comparison to control, Vγ9Vδ2-T cells treated by PA displayed a significant decrease in cytotoxic activity against K562 tumor cells while OA-treated Vγ9Vδ2-T cells showed an increase in cytotoxic activity against tumor cells, and OA could restore the diminished ability of Vγ9Vδ2-T cells to fight tumors caused by PA (Fig. 2a). We next determined how PA and OA influence the cytotoxic activity of Vγ9Vδ2-T cells toward various tumors. In line with the findings obtained from K562 cells, PA-treated Vγ9Vδ2-T cells exhibited greatly reduced cytotoxicity while OA-treated Vγ9Vδ2-T cells showed increased cytotoxicity toward nasopharyngeal cancer (HK-1), lung cancer (A549), cervical cancer (HeLa), breast cancer (MCF-7), ovarian cancer (A2780), and neuroblastoma (SK-N-BE2). Importantly, OA effectively restored the cytotoxic activity of PA-treated Vγ9Vδ2-T cells to a normal level (Fig. 2b). These findings demonstrated that PA dampened the capacity of Vγ9Vδ2-T cells in eliminating tumor cells while OA restored their antitumor activity in vitro.

To confirm the effect of PA and OA on the efficacy of Vγ9Vδ2-T cells against tumors, different diets conditioning Rag2^–/–^γc^–/–^ mice were established in which mice were fed with low-fat diet (LFD), palm oil high-fat diet (HFD), olive oil HFD, or palm and olive oil HFD for 30 days, and then GFP^+^ A549 tumor cells were inoculated into these mice subcutaneously. Subsequently, on day 7, 12, 17, and 22 following A549 cell inoculation, BSA-, PA-, OA- or PA + OA-treated Vγ9Vδ2-T cells were administered via intravenous injection into these mice, respectively (Fig. 2c). Custom HFDs were designed specifically for this experiment, with each diet containing a different fat source: 35% palm oil, 35% olive oil, or 35% palm and olive oil combined. The LFD did not contain palm oil or olive oil. The specially formulated diets for mice were consistent in their ingredients, differing only in the type and amount of fat included, ensuring that confounding effects typically present in human diets were avoided (Fig. 2d). Mice fed with LFD, palm oil HFD, olive oil HFD, and palm+olive oil HFD diets showed similar levels of metabolic parameters, which includes adiponectin, insulin, leptin, and resistin (Supplementary Fig. 2a). Without Vγ9Vδ2-T cells treatment, no differences in weight, tumor volume, or survival rate were found among the different diets-conditioning tumor-bearing mice (Supplementary Fig. 2c–g), indicating no direct impact of different diets on tumor growth in these mice during 60 days of observation. In the mice that received Vγ9Vδ2-T cells treatment, no difference in the weight changes was observed among the different diets conditioning mice (Supplementary Fig. 2b). Palm oil HFD-fed mice showed rapid tumor growth, and all these mice died within 44 days even after being treated with PA-treated Vγ9Vδ2-T cells compared to LFD-fed mice. Conversely, the tumor growth in olive oil HFD-fed mice was significantly inhibited and most of these mice survived until day 60 during treatment with OA-treated Vγ9Vδ2-T cells. Noticeably, a combination of PA and OA-treated Vγ9Vδ2-T cells effectively controlled tumor growth and extended the lifespan of the mice fed a palm + olive oil HFD (Fig. 2e–g). These findings indicated that OA could counteract the negative effects of PA and reverse the capacity of Vγ9Vδ2-T cells to combat cancers.

### OA rescued the reduced lytic granule secretion, glycolysis, and OXPHOS in Vγ9Vδ2-T cells induced by PA

**Fig. 3:**
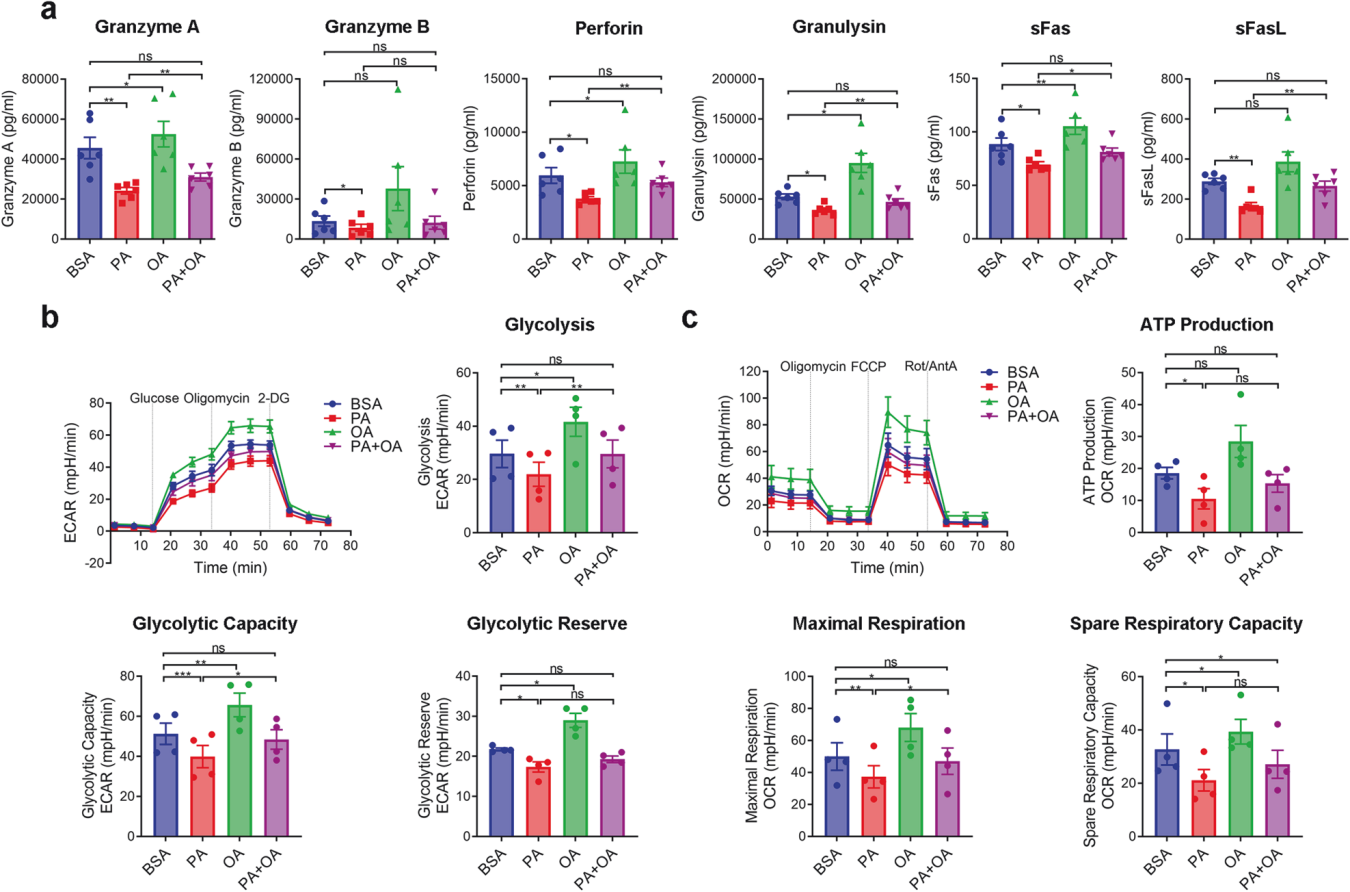
OA rescued the reduced lytic granule secretion, glycolysis and OXPHOS in Vγ9Vδ2-T cells induced by PA. **a** The secretions of granzyme A/B, perforin, granulysin, sFas and sFasL collected from the supernatant of the co-culturing system between BSA-, PA-, OA-, or PA + OA-treated Vγ9Vδ2-T cells and K562 tumor cells were measured (*n* = 6). **b**, **c** Real-time analysis of aerobic glycolysis (ECAR) and OXPHOS (OCR) in BSA-, PA-, OA-, PA + OA-Vγ9Vδ2-T cells were performed. **b** In the presence of oligomycin and 2-DG, ECAR curves were evaluated. Comparisons of glycolysis, glycolytic capacity, and glycolytic reserve are shown (*n* = 4). **c** After the supplement of oligomycin, FCCP, and rotenone/antimycin A, OCR curves were obtained. Comparisons of maximum respiration, ATP production, and spare respiration capacity, are shown (*n* = 4). The data are shown as the mean ± SEM. ns not significant; *p < 0.05; **p < 0.01; ***p < 0.001

The secretion of lytic granules and NKG2D activation are essential for Vγ9Vδ2-T cells to eliminate tumor cells directly.^21^ Therefore, lytic granules from PA-, OA-, or PA + OA-treated Vγ9Vδ2-T cells were evaluated during coculturing with tumor cells. Compared to control, the capacity of PA-treated Vγ9Vδ2-T cells in releasing granzyme A/B, perforin, granulysin, sFas, and sFasL was significantly reduced. In contrast, OA-treated Vγ9Vδ2-T cells enhanced most of lytic granule secretions. Importantly, OA effectively restored the reduction of these lytic granule secretions induced by PA (Fig. 3a).

Glycolysis and OXPHOS are essential processes to supply energy for immune cell activation and function. Therefore, we also analyzed the extracellular acidification rate (ECAR) and oxygen consumption rate (OCR), corresponding to glycolysis and OXPHOS. In comparison to the control, PA-treated Vγ9Vδ2-T cells exhibited a decrease in glycolysis, glycolytic capacity, and reserve. Conversely, OA treatment significantly increased their glycolysis, glycolytic capacity, and reserve (Fig. 3b). Similarly, PA decreased while OA increased ATP production, maximal respiration, and spare respiratory capacity in Vγ9Vδ2-T cells (Fig. 3c). OA restored the reduction of glycolysis and OXPHOS profiles in Vγ9Vδ2-T cells induced by PA (Fig. 3b, c). These results demonstrated that OA could rescue the diminished capacity of Vγ9Vδ2-T cells to combat tumors through reversing PA-induced reductions of lytic granules, glycolysis, and OXPHOS.

FAs are primarily metabolized through fatty acid oxidation (FAO) and restoring metabolic disorders in Vγ9Vδ2-T cells may reverse their cytotoxicity. We firstly detected the expression of some genes which are crucial in the process of FAO, including CPT1, CPT2, ACADL, HADHA, HADHB, and ECL. RT-qPCR analysis showed both PA and OA treatment upregulated the expression of these FAO-related genes in Vγ9Vδ2-T cells, especially the fatty acid translocase gene CPT1A (Supplementary Fig. 3a). To determine whether blocking fatty acid transport restores the cytotoxic function of Vγ9Vδ2-T cells toward tumors, etomoxir, the inhibitor of FAO through mainly inhibiting CPT1A, was used to block CPT1A for 24 h. CPT1A was inhibited after etomoxir treatment (Supplementary Fig. 3b). Etomoxir treatment significantly reversed the impaired capacity of Vγ9Vδ2-T cells to eliminate tumor cells induced by PA (Supplementary Fig. 3c). Consistent with these results, etomoxir treatment also restored decreased lytic granules secretion from PA-treated Vγ9Vδ2-T cells, including granzyme A, granzyme B, and granulysin (Supplementary Fig. 3d). Thus, restoring metabolic defects can reverse the impaired cytotoxic function of Vγ9Vδ2-T cells.

### OA restores Vγ9Vδ2-T cell antitumor activity by preventing PA-induced cell pyroptosis

**Fig. 4:**
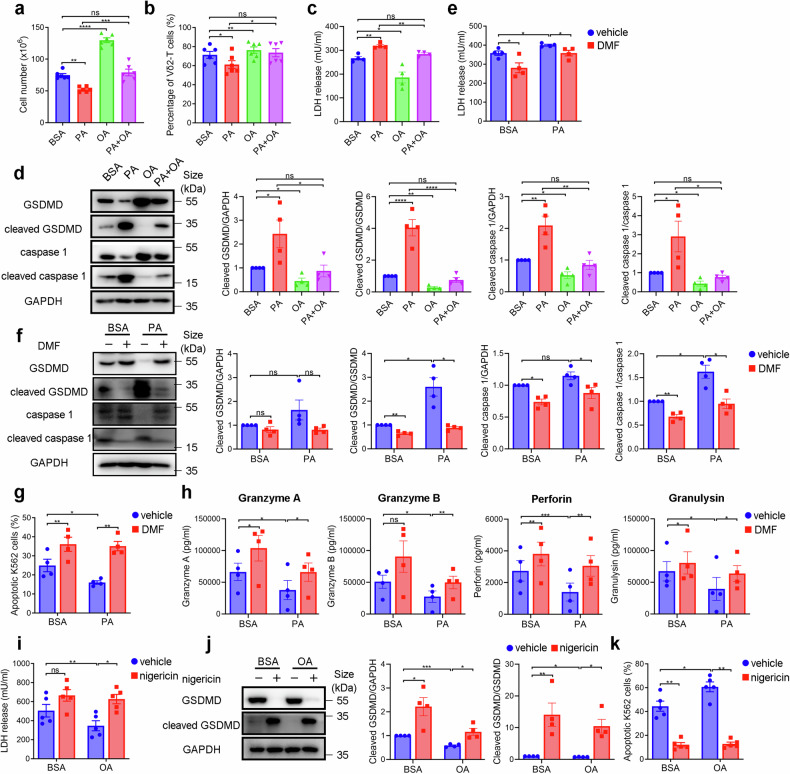
OA restores Vγ9Vδ2-T cell antitumor activity by preventing PA-induced cell pyroptosis. Vγ9Vδ2-T cells were expanded from PBMCs of healthy donors using PAM and IL-2 with the supplement with BSA, PA, OA, or a mixture of PA and OA for 14 days. The absolute cell numbers (**a**) and the percentages (**b**) of Vγ9Vδ2-T cells were examined (*n* = 6). **c** The level of LDH release in the culture medium of BSA-, PA-, OA-, or PA + OA-Vγ9Vδ2-T cells after culturing for 14 days was measured (*n* = 4). **d** Representative immunoblot (left) and quantification (right) of GSDMD, cleaved GSDMD, caspase-1, and cleaved caspase-1 in BSA-, PA-, OA-, or PA + OA -Vγ9Vδ2-T cells (*n* = 4). **e**–**g** Expanded BSA-, or PA- Vγ9Vδ2-T cells were treated with DMF to block pyroptosis. **e** The level of LDH release in the culture medium of BSA-, or PA -Vγ9Vδ2-T cells after DMF treatment overnight was measured (*n* = 4). **f** Representative immunoblot (left) and quantification (right) of GSDMD, cleaved GSDMD, caspase-1, and cleaved caspase-1 in BSA-, or PA -Vγ9Vδ2-T cells after DMF treatment overnight (n = 4). **g** The percentages of apoptotic tumor cells after coculture with BSA-, or PA-Vγ9Vδ2-T cells with or without DMF treatment were examined (*n* = 4). **h** The secretions of granzyme A/B, perforin, and granulysin collected from the supernatant of the co-culturing system after coculture BSA-, or PA -Vγ9Vδ2-T cells with tumor cells were examined (*n* = 4). **i**–**k** Expanded BSA-, or OA- Vγ9Vδ2-T cells were treated with nigericin to induce pyroptosis. **i** The level of LDH release in the culture medium of BSA-, or OA -Vγ9Vδ2-T cells after nigericin treatment overnight was measured (*n* = 5). **j** Representative immunoblot (left) and quantification (right) of GSDMD, and cleaved GSDMD in BSA-, or OA -Vγ9Vδ2-T cells after nigericin treatment overnight (*n* = 4). **k** The percentages of apoptotic tumor cells after coculture with BSA-, or OA-Vγ9Vδ2-T cells with or without nigericin treatment were examined (*n* = 5). The data are shown as the mean ± SEM. ns not significant; *p < 0.05; **p < 0.01; ***p < 0.001; ****p < 0.0001

FAs can induce lipotoxicity and consequently result in cell dysfunction. We thus examined Vγ9Vδ2-T cell viability after PA or OA treatment. As shown in Fig. 4a, b, PA increased cell death while OA enhanced cell growth and further rescued the cell death induced by PA, as evidenced by the alterations in the Vγ9Vδ2-T cell number and percentage after PA or OA treatment. To determine what type of cell death was induced by PA, we assessed cell apoptosis, ferroptosis, and pyroptosis. Neither PA nor OA could induce apoptotic and ferroptotic Vγ9Vδ2-T cells even after 14 days of exposure at current concentrations because no significant Annexin V^+^ and lipid ROS^+^ cells were detected (Supplementary Fig. 4a, b). Interestingly, we further found that PA markedly increased lactate dehydrogenase (LDH) release, a marker of cell damage, from Vγ9Vδ2-T cells. In contrast, OA decreased LDH released from Vγ9Vδ2-T cells (Fig. 4c). The mRNA expressions of prominent pyroptosis markers in Vγ9Vδ2-T cells, including GSDMD, ASC, and caspase 1, were detected after 14 days of exposure to PA or OA. PA upregulated the expression of GSDMD and caspase 1. In contrast, OA downregulated and further attenuated the impact of PA on the expressions of the three genes in Vγ9Vδ2-T cells (Supplementary Fig. 4c). The analysis of cleaved GSDMD and cleaved caspase 1 further confirmed that PA-induced cell pyroptosis while OA ameliorated PA-induced pyroptosis of Vγ9Vδ2-T cells (Fig. 4d). These data indicated that OA had a protective effect by mitigating the detrimental effects of PA-induced pyroptosis in Vγ9Vδ2-T cells.

Given that αβ T cells are the strongest components in the immune response against tumors and are the foundation of potent cancer immunotherapies,^22^ we assessed whether PA or OA affects the proliferation and cell death of CD4 and CD8 T cells. CFSE staining revealed that treatment with PA or OA has no impact on the proliferation of CD4 or CD8 T cells following CD3/CD28 stimulation (Supplementary Fig. 5a). No significant Annexin V^+^ and lipid ROS^+^ cells were detected in CD4 or CD8 T cells after 14 days of exposure under PA or OA treatment, indicating that PA or OA at current concentrations did not induce apoptosis or ferroptosis in CD4 or CD8 T cells (Supplementary Fig. 5b, c). Similarly, comparable levels of LDH release and cleaved GSDMD were observed in CD4 or CD8 cells treated with PA or OA in comparison to those treated with BSA (Supplementary Fig. 5d, e). These results indicated that PA induces cell pyroptosis in Vγ9Vδ2-T cells, not in CD4 or CD8 T cells.

To further evaluate whether the defective ability of Vγ9Vδ2-T cells against tumors prompted by PA is mediated by pyroptosis, DMF, an inhibitor of GSDMD,^23^ was utilized to treat Vγ9Vδ2-T cells under PA treatment. As expected, DMF inhibited PA-induced LDH release in Vγ9Vδ2-T cells (Fig. 4e). Similar inhibitory effects of DMF on the expression of cleaved GSDMD and cleaved caspase 1 were also observed in PA-treated Vγ9Vδ2-T cells (Fig. 4f). More importantly, the cytotoxicity of PA-treated Vγ9Vδ2-T cells toward tumor cells was reversed after DMF treatment (Fig. 4g). We also examined the lytic granules released from Vγ9Vδ2-T cells under BSA, or PA conditions when they were cocultured with tumor cells. DMF treatment reversed the decreased secretions of lytic granules induced by PA compared to control (Fig. 4h). In addition, the inducer of pyroptosis, nigericin, was used to treated Vγ9Vδ2-T cells under OA treatment. Similarly, nigericin attenuated the reduction of LDH release from OA-treated Vγ9Vδ2-T cells (Fig. 4i). The protein levels of cleaved GSDMD confirmed that nigericin-induced cell pyroptosis in OA-treated Vγ9Vδ2-T cells (Fig. 4j). Nigericin treatment significantly suppressed the ability of OA-treated Vγ9Vδ2-T cells in combating tumor cells (Fig. 4k). Taken together, our results demonstrated that PA-induced pyroptosis resulted in impaired Vγ9Vδ2-T cell cytotoxicity against tumor cells, and OA could restore their antitumor activity by preventing cell pyroptosis.

### IFNγ mediates PA-induced pyroptosis in Vγ9Vδ2-T cells

**Fig. 5:**
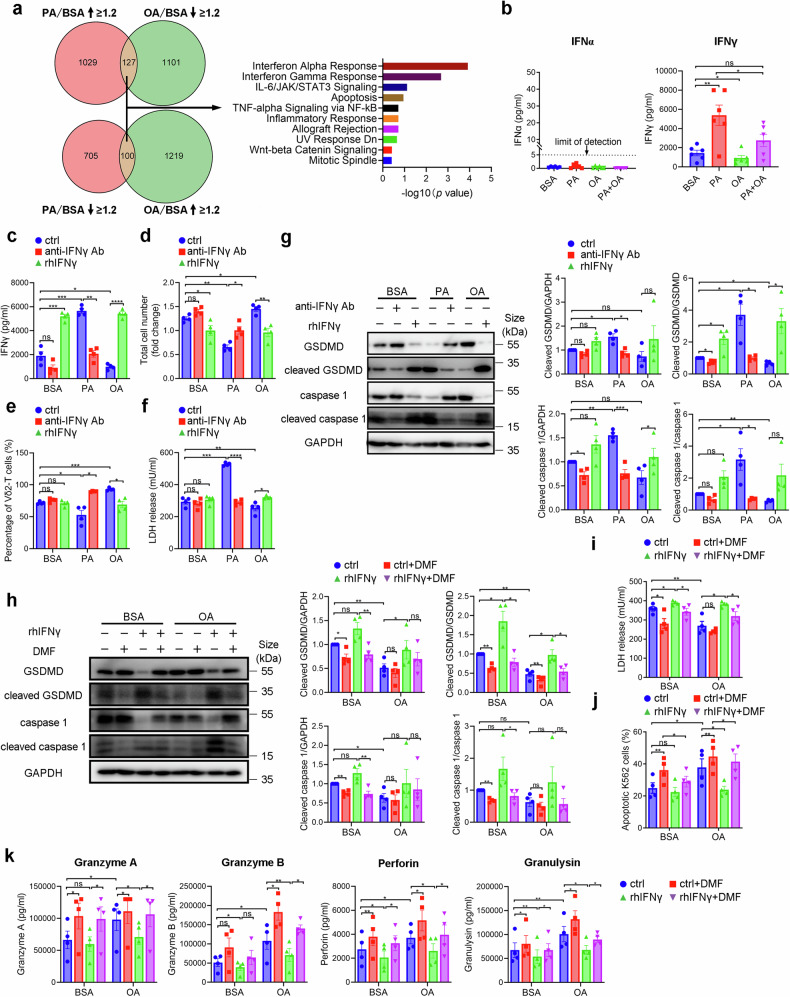
IFNγ mediates PA-induced pyroptosis in Vγ9Vδ2-T cells. **a** Proteomics analysis of cellular proteins from BSA-, PA-, or OA-Vγ9Vδ2-T cells after culturing for 14 days (*n* = 3 per group). Then pathway analysis of the distinguished proteins was performed in PA- and OA-treated Vγ9Vδ2-T cells compared to BSA-treated Vγ9Vδ2-T cells. **b** IFNα and IFNγ secretions collected from the supernatant of BSA-, PA-, OA-, or PA + OA-Vγ9Vδ2-T cells were measured by ELISA (*n* = 6). **c**–**g** Expanded BSA-, PA- or OA-Vγ9Vδ2-T cells were treated with or without anti-IFNγ Ab or rhIFNγ for 3 days. **c** The secretion of IFNγ collected from the supernatant of Vγ9Vδ2-T cells was measured by ELISA (*n* = 4). The absolute cell numbers (**d**) and the percentages (**e**) of Vγ9Vδ2-T cells were examined (*n* = 4). **f** The level of LDH release in the culture medium of Vγ9Vδ2-T cells was measured (*n* = 4). **g** Representative immunoblot (left) and quantification (right) of GSDMD, cleaved GSDMD, caspase-1 and cleaved caspase-1 in Vγ9Vδ2-T cells (*n* = 4). **h**–**k** Expanded BSA-, or OA- Vγ9Vδ2-T cells were treated with rhIFNγ for 3 days, and then DMF was used to block pyroptosis. **h** Representative immunoblot (left) and quantification (right) of GSDMD, cleaved GSDMD, caspase-1 and cleaved caspase-1 in BSA-, or OA-Vγ9Vδ2-T cells (*n* = 4). **i** The level of LDH release in the culture medium of BSA-, or OA-Vγ9Vδ2-T cells was measured (*n* = 4). **j** The percentages of apoptotic tumor cells after coculture with BSA-, or OA-Vγ9Vδ2-T cells were examined (*n* = 4). **k** The secretions of granzyme A/B, perforin, and granulysin collected from the supernatant of the co-culturing system after coculture BSA-, or OA -Vγ9Vδ2-T cells with tumor cells were examined (*n* = 4). The data are shown as the mean ± SEM. ns not significant; *p < 0.05; **p < 0.01; ***p < 0.001; ****p < 0.0001

To better understand the mechanisms underlying the different antitumor activity of Vγ9Vδ2-T cells induced by PA or OA, we performed the proteomic analysis of Vγ9Vδ2-T cells cultured under different conditions. The Venn diagram showed the number of proteins differentially expressed on Vγ9Vδ2-T cells under PA or OA conditions with more than 1.2 times of fold change. Enrichment pathway analysis revealed that the top two correlated pathways were IFNα and IFNγ responses (Fig. 5a). We then measured the concentrations of IFNα and IFNγ in the supernatant from Vγ9Vδ2-T cells. The secretion of IFNα from Vγ9Vδ2-T cells was too low to measure. Interestingly, PA-treated Vγ9Vδ2-T cells had an increase of IFNγ secretion while OA-treated cells had a decrease of IFNγ secretion in their culture supernatants (Fig. 5b). Similar results were also observed in the supernatants when Vγ9Vδ2-T cells cocultured with tumor cells (Supplementary Fig. 6). It has been proven that TNFα and IFNγ can cause inflammatory cell death, PANoptosis.^24^ To determine the influence of IFNγ in pyroptosis of Vγ9Vδ2-T cells, anti-IFNγ neutralizing Ab or recombinant human IFNγ (rhIFNγ) were added during culturing Vγ9Vδ2-T cells (Fig. 5c). The decrease in cell number and percentage of Vγ9Vδ2-T cells treated with PA was reversed upon IFNγ blockade, whereas under OA conditions, supplementation with rhIFNγ led to a decrease in both Vγ9Vδ2-T cell number and percentage (Fig. 5d, e). Blockade of IFNγ also significantly reduced LDH release from PA-treated Vγ9Vδ2-T cells, whereas rhIFNγ treatment increased LDH release from OA-treated Vγ9Vδ2-T cells (Fig. 5f). These data indicated that IFNγ participated in Vγ9Vδ2-T cell death induced by PA.

We next sought to determine whether IFNγ could mediate pyroptosis induced by PA. When IFNγ was blocked, protein levels of pyroptosis marker, cleaved GSDMD, and cleaved caspase 1, were significantly inhibited under PA treatment. Conversely, the supplement of rhIFNγ increased the expressions of these proteins on Vγ9Vδ2-T cells under OA conditions (Fig. 5g). Our findings demonstrated that IFNγ mediated PA-induced pyroptosis of Vγ9Vδ2-T cells.

Given that the pyroptosis had a critical effect on the cytotoxicity of Vγ9Vδ2-T cell against tumor cells, we next determined whether the pyroptosis induced by rhIFNγ can cause the impaired antitumor ability. Due to less pyroptosis in OA-treated Vγ9Vδ2-T cells, we supplemented rhIFNγ in OA-treated Vγ9Vδ2-T cells to establish the pyroptosis model generated by rhIFNγ. We firstly confirmed that DMF treatment effectively inhibited enhanced GSDMD cleavage and caspase 1 activation of Vγ9Vδ2-T cells after rhIFNγ stimulation under BSA or OA conditions (Fig. 5h). Further, there was decreased LDH release from OA- treated Vγ9Vδ2-T cells with the supplement of rhIFNγ after DMF treatment compared with the vehicle group (Fig. 5i). In addition, K562 tumor cells had moderate resistance to the killing of Vγ9Vδ2-T cells treated with rhIFNγ and DMF treatment alleviated the dampened cytotoxic activity of Vγ9Vδ2-T cells prompted by rhIFNγ (Fig. 5j). Similarly, DMF treatment restored the reduced secretions of lytic granules from OA-treated Vγ9Vδ2-T cells induced by rhIFNγ supplementation (Fig. 5k). We also demonstrated that DMF treatment rescued the excessive secretion of IFNγ from Vγ9Vδ2-T cells induced by PA to the normal level (Supplementary Fig. 7). Collectively, these findings verified that IFNγ mediated PA-induced Vγ9Vδ2-T cell pyroptosis and targeting IFNγ could regulate the cytotoxic capacity of Vγ9Vδ2-T cells in eliminating tumors.

### *p*STAT1-IRF1-iNOS axis mediates IFNγ-induced pyroptosis in Vγ9Vδ2-T cells

**Fig. 6:**
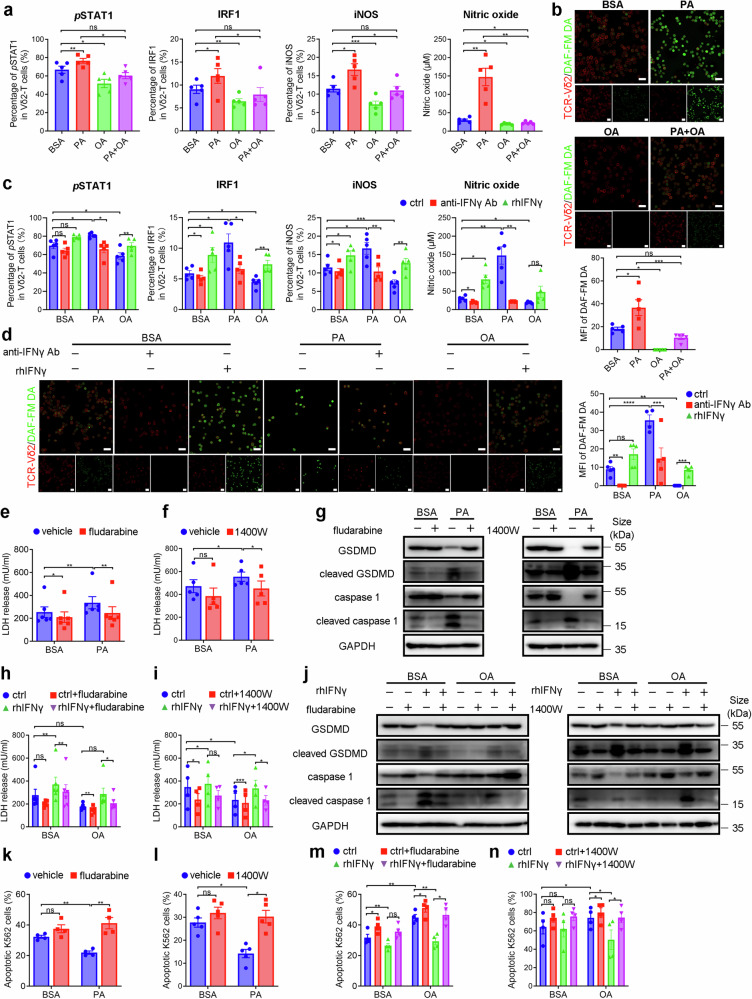
*p*STAT1-IRF1-iNOS pathway mediates IFNγ-induced pyroptosis in Vγ9Vδ2-T cells. **a**, **b** Vγ9Vδ2-T cells were cultured with BSA, PA, OA, or a mixture of PA and OA for 14 days. **a** *p*STAT1, IRF1, iNOS expression on Vγ9Vδ2-T cells, and NO level in their supernatants were examined (*n* = 5). **b** The NO level in Vγ9Vδ2-T cells was quantified using DAF-FM DA probe under confocal microscopy. Representative confocal images and quantification of average fluorescence intensity are shown (*n* = 5). The scale bar represents 20 µm. **c**, **d** Expanded BSA-, PA- or OA-Vγ9Vδ2-T cells were treated with or without anti-IFNγ Ab or rhIFNγ. **c** *p*STAT1, IRF1, and iNOS expression on Vγ9Vδ2-T cells and NO level in their supernatants were examined (*n* = 5). **d** The NO level in Vγ9Vδ2-T cells was quantified using DAF-FM DA probe under confocal microscopy. Representative confocal images and quantification of average fluorescence intensity are shown (*n* = 5). The scale bar represents 20 µm. The level of LDH release in the culture medium of BSA-, or PA-Vγ9Vδ2-T cells after fludarabine (**e**) or 1400 W (**f**) treatment was measured (*n* = 5). **g** Representative immunoblot of GSDMD, cleaved GSDMD, caspase-1, and cleaved caspase-1 in BSA-, or PA-Vγ9Vδ2-T cells after fludarabine (left) or 1400 W (right) treatment. **h**–**j** Expanded BSA-, or OA- Vγ9Vδ2-T cells were treated with fludarabine or 1400 W, then rhIFNγ was used. **h**, **i** The level of LDH release in the culture medium of BSA-, or OA-Vγ9Vδ2-T cells was measured (*n* = 6). **j** Representative immunoblot of GSDMD, cleaved GSDMD, caspase-1 and cleaved caspase-1 in BSA-, or OA-Vγ9Vδ2-T cells after fludarabine (left) or 1400 W (right) treatment. The percentages of apoptotic tumor cells after coculture with BSA-, or PA-Vγ9Vδ2-T cells with or without fludarabine (**k**) or 1400 W (**l**) treatment were examined (*n* = 4). The percentages of apoptotic tumor cells after coculture with BSA-, or OA-Vγ9Vδ2-T cells in the presence of rhIFNγ with or without fludarabine (**m**) or 1400 W (**n**) treatment were examined (*n* = 4). The data are shown as the mean ± SEM. ns not significant; *p < 0.05; **p < 0.01; ***p < 0.001; ****p < 0.0001

We next determine the mechanisms underlying IFNγ-induced pyroptosis in Vγ9Vδ2-T cells. STAT1-IRF1-iNOS axis was found to mediate TNFα and IFNγ-induced PANoptosis.^24^ In comparison to control, PA significantly upregulated *p*STAT1, IRF1, and iNOS levels in the Vγ9Vδ2-T cells, along with elevated nitric oxide (NO) levels in their supernatants and increased NO signals in these cells. While OA downregulated the level of these markers and reversed their expression on Vγ9Vδ2-T cells induced by PA (Fig. 6a, b and Supplementary Fig. 8a). Blockade of IFNγ in PA-treated Vγ9Vδ2-T cells significantly decreased their expression while supplementary of rhIFNγ in OA-treated Vγ9Vδ2-T cells increased their expression (Fig. 6c, d and Supplementary Fig. 8b). To evaluate whether *p*STAT1, IRF1, iNOS, and NO mediates IFNγ-induced pyroptosis in PA-Vγ9Vδ2-T cells, we utilized fludarabine and 1400 W, the inhibitors of STAT1 phosphorylation and NO synthesis respectively, to block this pathway. As expected, fludarabine significantly inhibited *p*STAT1 level and NO signals in the Vγ9Vδ2-T cells (Supplementary Fig. 8c, d). 1400 W also inhibited the levels of NO signals in the Vγ9Vδ2-T cells and NO release in their supernatants (Supplementary Fig. 8e, f). Treatment with fludarabine or 1400 W inhibited PA-induced LDH release in Vγ9Vδ2-T cells (Fig. 6e, f). Similar inhibitory effects of fludarabine or 1400 W on the expression of cleaved GSDMD and cleaved caspase 1 were also observed in PA-treated Vγ9Vδ2-T cells (Fig. 6g). Moreover, inhibition of fludarabine was also confirmed by the decreased expression of *p*STAT1 and NO signals in the Vγ9Vδ2-T cells after rhIFNγ treatment (Supplementary Fig. 8g, h). 1400 W also inhibited the elevated levels of NO signals in the Vγ9Vδ2-T cells and increased NO release in their supernatants induced by rhIFNγ (Supplementary Fig. 8i, j). Furthermore, there was decreased LDH release from OA-treated Vγ9Vδ2-T cells with the supplement of rhIFNγ after fludarabine or 1400 W treatment compared to those in the vehicle group (Fig. 6h, i). Fludarabine or 1400 W treatment effectively suppressed the induction of GSDMD cleavage and caspase 1 activation of Vγ9Vδ2-T cells following rhIFNγ stimulation under BSA or OA conditions (Fig. 6j). More importantly, the cytotoxicity of PA-treated Vγ9Vδ2-T cells toward tumor cells was reversed after fludarabine or 1400 W treatment (Fig. 6k, l). In addition, K562 tumor cells had moderate resistance to the killing of Vγ9Vδ2-T cells in the presence of rhIFNγ and inhibition of STAT1 activation and NO synthesis by fludarabine or 1400 W alleviated the dampened cytotoxic capacity of Vγ9Vδ2-T cells prompted by rhIFNγ (Fig. 6m, n). Taken together, these data demonstrated that *p*STAT1-IRF1-iNOS axis mediated IFNγ-induced pyroptosis in Vγ9Vδ2-T cells.

### IFNγ mediates PA-induced functional and metabolic defects in Vγ9Vδ2-T cells

**Fig. 7:**
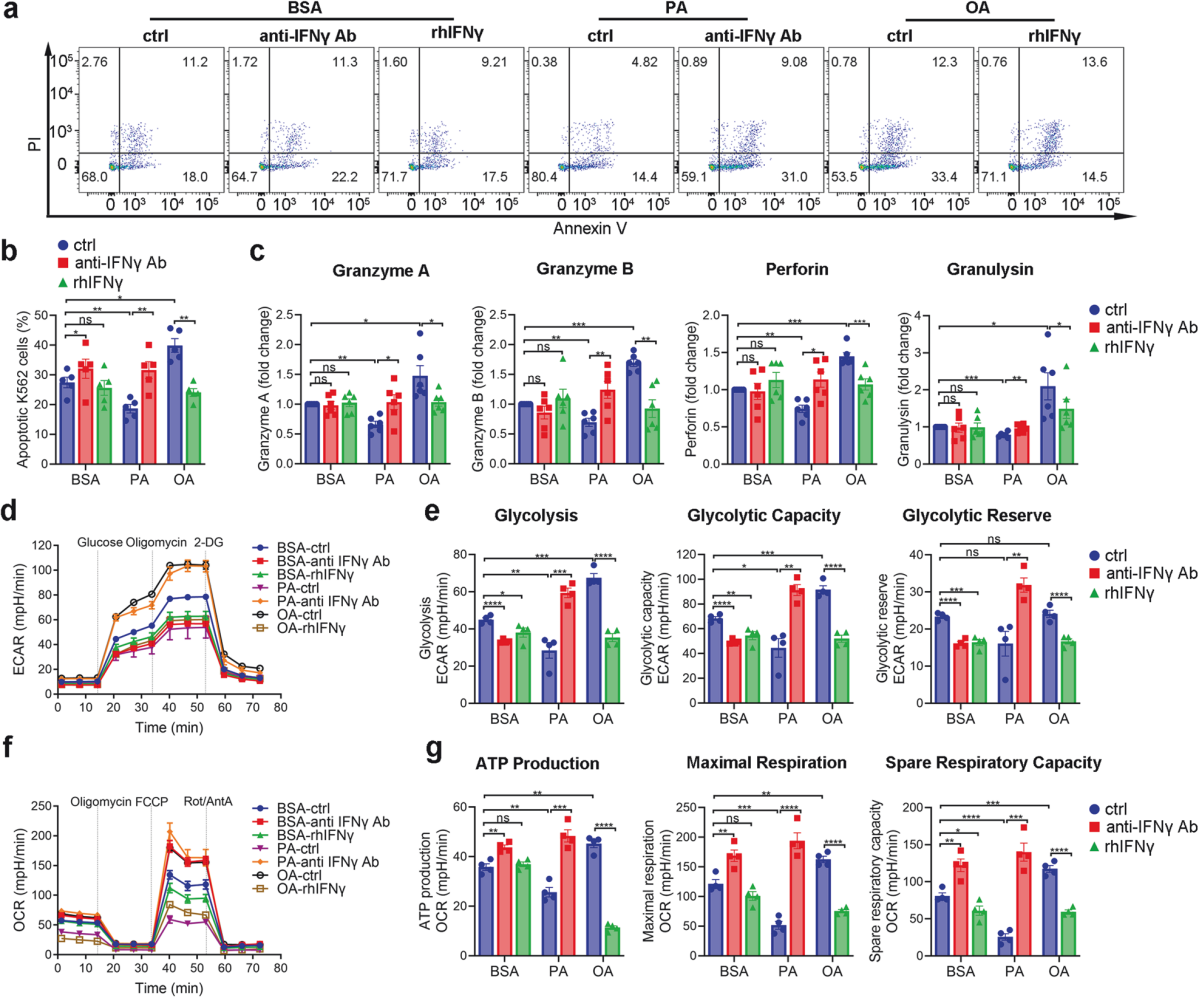
IFNγ mediates PA-induced functional and metabolic defects in Vγ9Vδ2-T cells. **a** The antitumor activity of expanded BSA-, PA- or OA-Vγ9Vδ2-T cells with or without anti-IFNγ Ab or rhIFNγ was detected by co-culturing Vγ9Vδ2-T cells with K562 tumor cells at an effector/target cells of 10:1 for 6 h, and then the apoptotic K562 cells were determined by flow cytometry. **b** Quantitative comparisons of apoptotic K562 cells are shown (*n* = 5). **c** The secretions of granzyme A/B, perforin and granulysin collected from the supernatant of the co-culturing system between expanded BSA-, PA- or OA-Vγ9Vδ2-T cells with or without anti-IFNγ Ab or rhIFNγ and K562 tumor cells were measured (*n* = 6). **d**–**g** Real-time analysis of aerobic glycolysis (ECAR) and OXPHOS (OCR) in expanded BSA-, PA- or OA-Vγ9Vδ2-T cells with or without anti-IFNγ Ab or rhIFNγ was performed. **d** In the presence of oligomycin and 2-DG, ECAR curves were assessed. **e** Quantitative comparisons of glycolysis, glycolytic capacity, and glycolytic reserve, are shown (*n* = 4). **f** After the supplement of oligomycin, FCCP, and rotenone/antimycin A, OCR curves were obtained. **g** Quantitative comparisons of maximum respiration, ATP production, and spare respiration capacity, are shown (*n* = 4). The data are shown as the mean ± SEM. ns not significant; *p < 0.05; **p < 0.01; ***p < 0.001; ****p < 0.0001

To determine the role of IFNγ in the function and metabolic profiles in Vγ9Vδ2-T cells, anti-IFNγ neutralizing mAb or rhIFNγ was used in PA- or OA-treated Vγ9Vδ2-T cells. Blockade of IFNγ in PA-treated Vγ9Vδ2-T cells significantly enhanced their antitumor activity, while supplementary of rhIFNγ in OA-treated Vγ9Vδ2-T cells decreased their antitumor activity (Fig. 7a, b). We next sought to evaluate whether IFNγ influences the lytic secretion and metabolic profile of Vγ9Vδ2-T cells. Blockade of IFNγ significantly increased granzyme A, granzyme B, perforin and granulysin secretions in PA-treated Vγ9Vδ2-T cells while rhIFNγ supplementation reduced these lytic granules secretions in OA-treated Vγ9Vδ2-T cells compared to BSA group (Fig. 7c). Furthermore, glycolysis, glycolytic capacity and reserve were significantly increased in PA-treated Vγ9Vδ2-T cells when IFNγ was neutralized. In contrast, these parameters were significantly reduced in OA-treated Vγ9Vδ2-T cells after the addition of rhIFNγ (Fig. 7d, e). Moreover, the PA-induced OCR, including ATP production, maximal respiration, and spare respiratory capacity was significantly increased in Vγ9Vδ2-T cells after anti-IFNγ neutralizing mAb was used. In contrast, OA-induced OCR was decreased in Vγ9Vδ2-T cells after the supplementation of rhIFNγ (Fig. 7f, g). These results demonstrated that excess IFNγ could inhibit the secretion of lytic granules and induce metabolic defects, subsequently leading to impaired Vγ9Vδ2-T cell functions.

### Blockade of IFNγ and pyroptosis restores PA-impaired antitumor activity of Vγ9Vδ2-T cells in vivo

**Fig. 8:**
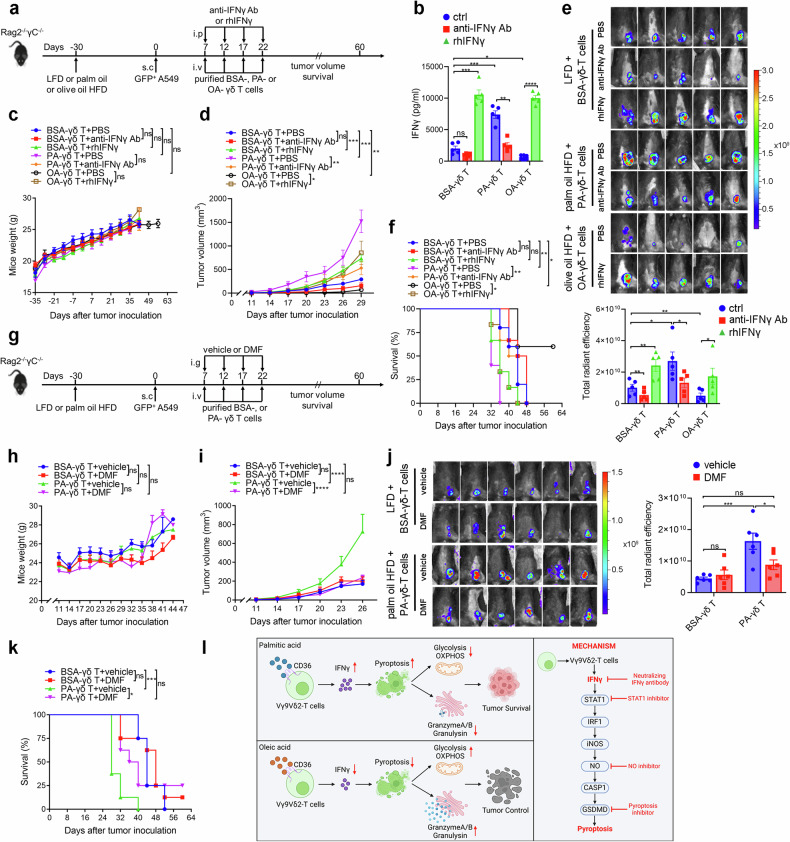
Blockade of IFNγ and pyroptosis restores PA-impaired antitumor activity of Vγ9Vδ2-T cells in vivo. **a** Diagram of the protocol in (**b**–**f**). Rag2^−/−^γc^−/−^ mice fed on the LFD, palm oil, or olive oil HFDs for 30 days were subcutaneously (s.c.) injected with GFP^+^ A549 tumor cells. Then anti-IFNγ Ab or rhIFNγ was intraperitoneally (i.p.) injected into mice. Expanded BSA-, PA-, or OA-Vγ9Vδ2-T cells were intravenously (i.v.) transferred into the mice at the indicated time (*n* = 5 mice per group). **b** The level of IFNγ from mice serum collected on the 2^nd^ day after injection of anti-IFNγ Ab or rhIFNγ was measured. **c** Mouse weight was monitored for 2 months. **d** Tumor volumes were obtained at the indicated time. **e** On day 29 following GFP^+^ A549 tumor cells inoculation, whole-body fluorescence images (upper) and total radiant efficiency of fluorescence intensity (lower) of the mice are shown after Vγ9Vδ2-T cell treatment. **f** Survival curves were obtained at the indicated time. **g** Diagram of the protocol in (**h**–**k**). Rag2^−/−^γc^−/−^ mice fed on the LFD, or palm oil HFD for 30 days were subcutaneously (s.c.) injected with GFP^+^ A549 tumor cells. Then vehicle or DMF was intragastrically (i.g.) injected into mice. Expanded BSA-, or PA-Vγ9Vδ2-T cells were intravenously (i.v.) transferred into the mice at the indicated time (*n* = 6 mice per group). **h** Mouse weight was monitored at the indicated time. **i** Tumor volumes were obtained at the indicated time. **j** On day 29 following GFP^+^ A549 tumor cells inoculation, whole-body fluorescence images (upper) and total radiant efficiency of fluorescence intensity (lower) of the mice were assessed after Vγ9Vδ2-T cell treatment. **k** Survival curves were acquired at the specified time. **l** Schematic overview of the effect of PA and OA on the antitumor activity and the mechanism of IFNγ-induced pyroptosis in Vγ9Vδ2-T cells. The schematic was made using Biorender and included a publication license (Zhang (2025) https://BioRender.com/h90t444). Quantitative data are shown as the mean ± SEM. ns not significant; *p < 0.05; **p < 0.01; ***p < 0.001; ****p < 0.0001

Then the effect of IFNγ on the antitumor ability of Vγ9Vδ2-T cells in the Rag2^–/–^γc^–/–^ mice fed with various diets was investigated. Rag2^–/–^γc^–/–^ mice were fed with LFD, palm oil HFD, or olive oil HFD for 30 days. Then GFP^+^ A549 tumor cells were subcutaneously inoculated into these mice. On days 7, 12, 17, and 22 following A549 cells inoculation, human anti-IFNγ neutralizing mAb or rhIFNγ was injected into mice intraperitoneally, and after 12 h, BSA-, PA-, or OA-treated Vγ9Vδ2-T cells were administered intravenously to tumor-bearing mice, respectively (Fig. 8a). The efficacy of neutralizing anti-IFNγ mAb or rhIFNγ in vivo was confirmed as evidenced by the reduced level of IFNγ or increased level of IFNγ in mouse serum after treatments, respectively (Fig. 8b). Neither anti-IFNγ mAb nor rhIFNγ affects mouse weight (Fig. 8c). In consistent with previous results (Fig. 2e), tumor growth was increased in mice fed on the palm oil HFD with PA-Vγ9Vδ2-T cells treatment while tumor growth was inhibited in mice fed on the olive oil HFD with OA-Vγ9Vδ2-T cells treatment (Fig. 8d). PA-Vγ9Vδ2-T cells showed a significantly increase in killing tumor cells when IFNγ was neutralized in mice fed on the palm oil HFD. Conversely, the antitumor ability of OA-Vγ9Vδ2-T cells was significantly inhibited after the supplementation of rhIFNγ in mice fed on the olive oil HFD (Fig. 8d, e). Blockade of IFNγ also prolonged the survival of mice fed on the palm oil HFD with PA-Vγ9Vδ2-T cells treatment while supplementation of IFNγ reduced the life span of mice fed on the olive oil HFD with OA-Vγ9Vδ2-T cells treatment (Fig. 8f). To exclude the potential direct impact of IFNγ on the tumor growth, after Rag2^–/–^γc^–/–^ mice were fed on LFD, palm oil HFD, or olive oil HFD for 30 days, the mice received an intraperitoneal injection of either anti-IFNγ mAb or rhIFNγ after 7 days of GFP^+^ A549 tumor cells inoculation (Supplementary Fig. 9a). Since there was no Vγ9Vδ2-T cells injection, the level of IFNγ was extremely low in mouse serum, and only the mice injected with rhIFNγ had a high level of IFNγ in the serum (Supplementary Fig. 9b). As shown in Supplementary Fig. 9c, neither human anti-IFNγ antibody nor rhIFNγ has a direct influence in the tumor growth. Taken together, these results confirmed that IFNγ mediated the impaired antitumor activity of Vγ9Vδ2-T cells induced by PA in vivo.

To further explore the effect of pyroptosis on the capacity of Vγ9Vδ2-T cells to combat tumors, mice were fed with LFD, or palm oil HFD for 30 days. Then the GFP^+^ A549 tumor cells were subcutaneously inoculated into these mice. On day 7, 12, 17, and 22 following A549 cells inoculation, DMF was intragastrically injected into mice, and after 12 h, the tumor-bearing mice received intravenous injections of BSA or PA-treated Vγ9Vδ2-T cells, respectively (Fig. 8g). DMF treatment had no impact on mouse weight (Fig. 8h). Importantly, we found that DMF treatment significantly enhanced the ability of PA-treated Vγ9Vδ2-T cells to inhibit tumor growth in mice fed a palm oil HFD (Fig. 8i, j). Blockade of pyroptosis by DMF also prolonged the survival of mice fed on the palm oil HFD with PA-Vγ9Vδ2-T cells treatment (Fig. 8k). These results further demonstrated that pyroptosis mediated PA-induced decreased capacity of Vγ9Vδ2-T cells to eliminate tumors in vivo.

## Discussion

Obesity is increasingly recognized to result in immunological dysfunction,^25^ but how individual FAs in human diets affect the function of immune cells is less known. In a group of cancer patients with Vγ9Vδ2-T cell therapy, we showed that the level of serum OA and the ratio of PA/OA were connected to therapeutic outcome of Vγ9Vδ2-T cells. Using high PA or OA to mimic various conditions that are richer in FAs in human body, here we proved that PA inhibited the antitumor efficacy of Vγ9Vδ2-T cells by impeding lytic granules secretions and reducing glycolysis and OXPHOS. We further clarified a novel underlying mechanism linked to the functional defects of Vγ9Vδ2-T cells caused by PA, whereby PA stimulated Vγ9Vδ2-T cells to secret excess IFNγ, which in turn induced cell pyroptosis, caused functional and metabolic defects, and ultimately led to the defective capacity of Vγ9Vδ2-T cells against tumor cells. Importantly, we demonstrated that OA could restore all these abnormalities induced by PA. In addition, direct blockade of IFNγ by anti-IFNγ mAb or inhibition of pyroptosis by DMF also restores their antitumor activity. Our study suggests that targeting IFNγ-mediated pyroptosis could reverse PA-induced downregulated antitumor immunity of γδ-T cells and dietary OA supplementation might improve their responsiveness to Vγ9Vδ2-T cell-based immunotherapies.

Diet influences numerous diseases, including cancers since the nutrient composition of the environment where immune cells and tumor cells survive was changed. Our previous study also found that high glucose impaired anticancer activity of Vγ9Vδ2-T cells, which was reversed through metformin treatment or glucose control.^21^ Despite numerous clinical trials investigating the impact of specific FAs on breast cancer risk, definitive conclusions remain elusive due to the presence of multiple confounding factors in human dietary patterns.^26^ It is generally accepted that unsaturated FAs may be beneficial while saturated FAs may be harmful to health.^27^ Pascual et al. showed that PA significantly increased metastatic lesion number and size in oral carcinoma and melanoma models compared to OA or linoleic acid.^28^ Additionally, PA was found to enhance the expression of CD36, a fatty acid transporter, thereby intensifying tumor cell invasiveness.^28^ Conversely, OA generally exhibits neutral or inhibitory effects on tumor growth.^29^ In our study, we observed that neither PA nor OA alone had a significant influence on A549 tumor growth in our model over the 60-day observation period. Importantly, here we discovered that PA inhibited the antitumor activity of Vγ9Vδ2-T cells while OA could restore their decreased antitumor activity induced by PA in vitro and in vivo. In support of our data, Sun et al. also found that PA accelerated tumor growth by increasing Tregs population and αβ-T cell exhaustion in TME, whereas OA rescued the impaired αβ-T cell antitumor immunity.^30^ Therefore, PA should be avoided while OA supplementation should be recommended in their diets for cancer patients to improve clinical outcomes of γδ-T cell-based therapy.

The immune system of the organism can be affected by the metabolic state through modifying energy substrates and metabolic processes in immune cells.^31^ Previously, we demonstrated that dysregulated glucose metabolism impaired the antitumor activity of Vγ9Vδ2-T cells.^21^ FAs are primarily catabolized by FAO, a process that needs the transport of lipids into the mitochondria by the rate-limiting enzyme CPT1.^32^ Inhibiting FA transport can trigger a metabolic shift from OXPHOS to glycolysis.^33^ Previous research has demonstrated that glycolysis is crucial for the effector functions of immune cells.^34^ Here we showed that PA could upregulate the expression of CPT1A in Vγ9Vδ2-T cells. Despite defects in glycolysis and OXPHOS in PA-treated Vγ9Vδ2-T cells, blockade of CPT1 by etomoxir in Vγ9Vδ2-T cells could restore their impaired antitumor function induced by PA (Supplementary Fig. 3). Our observation also aligns with a study by Michelet et al., who demonstrated that blocking lipid transport into mitochondria reversed metabolic paralysis in NK cells, subsequently restoring their cytotoxicity against tumor cells.^35^ Therefore, reversing the metabolic defects associated with lipid uptake by blocking CPT1, could allow Vγ9Vδ2-T cells to regain their cytotoxic capabilities. Our study also emphasizes the significant impact of the metabolic state on immune cell function, particularly the availability and metabolism of FAs.

Besides studying the interaction between FAs and Vγ9Vδ2-T cells, here the influence of FAs in the αβ-T cells was also investigated. Different from that on Vγ9Vδ2-T cells, no significant impact on the proliferation and survival of αβ-T cells was observed after being treated with PA or OA at the same dose and exposure time to Vγ9Vδ2-T cells (Supplementary Fig. 5). In line with our data, by using murine mammary tumor models, Rong Jin et al. demonstrated that OA did not induce αβ-T cell death.^36^ Hu et al. also demonstrated that the use of PA at doses below 400 µM did not influence CD8 T cell activation and proliferation.^37^ Taken together, our studies imply that αβ-T cells might be less vulnerable to FAs than Vγ9Vδ2-T cells. The data obtained from the current study that Vγ9Vδ2-T cells expressed higher FA transporter CD36 than CD4 or CD8 T cells may also support this.

Both pyroptosis and apoptosis belong to programmed death pathways. Pyroptosis is triggered through GSDMD cleavage by caspase-1 via inflammatory signaling.^38^ Pyroptosis is linked to proinflammatory cytokines secretion and induces inflammation, whereas apoptosis is primarily involved in maintaining tissue homeostasis and development and eliminating damaged or unwanted cells without inducing inflammation. Previous studies showed that PA induced both pyroptosis and apoptosis in certain cell types.^39^ The specific effects of PA on cell death are dependent on the concentration, duration of exposure, and the presence of other stimuli or inflammatory mediators. However, whether PA can induce T-cell pyroptosis remains unexplored. In the current study, our findings revealed that PA-induced Vγ9Vδ2-T cell apoptosis when the exposure dose of PA reached 100 µM, whereas OA was unable to induce apoptosis even at the dose of 400 µM. Interestingly, when reducing PA to a dose at 50 µM cannot cause cell apoptosis, significant Vγ9Vδ2-T cell pyroptosis was still found (Supplementary Fig. 4a), indicating that Vγ9Vδ2-T cells are easier to pyroptosis than apoptosis after exposure to PA. In our study, we demonstrated that blocking pyroptosis with DMF in the PA-treated Vγ9Vδ2-T cells or OA-treated Vγ9Vδ2-T cells in the presence of rhIFNγ restores decreased secretions of lytic granules induced by PA or rhIFNγ. However, studies indicate that pyroptosis results in cell membrane perforation and IL-1β and IL-18 release. The disparity in lytic granule levels and inflammatory cytokines may be attributed to the fact that while GSDMD pores facilitate IL-1β release, excessive membrane damage could lead to rapid cell disintegration before granzyme-containing granules can be adequately exocytosed.^40^ The process of granzyme secretion necessitates precise vesicle fusion, which may be disrupted by premature membrane rupture during pyroptosis. Additionally, PA could potentially drive T cells toward a pro-inflammatory state that prioritizes IL-1β secretion over cytotoxic function.

Based on different types of tumors, the stage of tumor development, and specific TME, IFNγ exerts a complex influence on tumor development and progression.^41^ Except for its antitumor effects widely described before, the pro-tumor effects of IFNγ have been paid more attention during recent years. IFNγ may promote tumor metastasis by inducing the upregulation of ICAM1, CD133, and CXCR4, and the production of MUC4, as well as promoting EMT.^42,43^ IFNγ may also increase indoleamine-2,3-dioxygenase (IDO), CTLA4, and PD-L1 production, thus mediating tumor immune escape.^44,45^ In the current study, we provide a novel mechanism involved in its pro-tumor effects, whereby PA stimulates Vγ9Vδ2-T cells to secret an excessive amount of IFNγ, which further induces Vγ9Vδ2-T cell pyroptosis and impairs their function and metabolism, ultimately leading to the loss of their antitumor activity. Importantly, here we also demonstrated that blockade of IFNγ can prevent PA-induced Vγ9Vδ2-T cell pyroptosis and restore their antitumor activity in vitro and in vivo. Moreover, our research elucidated the molecular mechanism mediated by IFNγ to promote cell death, identifying several novel potential therapeutic targets. We uncovered a pivotal function for STAT1 and NO downstream of IFNγ in driving pyroptosis. Other studies reveal that STAT1 can modulate cell death through inducing cell death dependent on caspase 8 and TNFα.^46^ We further elucidated the significance of iNOS and NO downstream of STAT1 and IRF1 in the pyroptotic cascade. The dual nature of NO as a cytotoxic or cytostatic agent has been well-documented in various contexts,^47^ with some studies indicating its capability to impede NLRP3 inflammasome assembly.^48^ Notably, NO has been implicated in involving pyroptosis^49^ and inducing T cell death following the release of IFNγ.^50^ Moreover, T cells lacking STAT1 and iNOS have reduced activation-induced cell death.^51^ Therefore, preventing excessive IFNγ secretion or targeting these common steps downstream of IFNγ may have the potential to maintain Vγ9Vδ2-T cell survival and enhance their antitumor activity.

Of note, here we found that prevention of pyroptosis by DMF resulted in a significant decrease of IFNγ secretion from PA-treated Vγ9Vδ2-T cells (Supplementary Fig. 7), suggesting a positive feedback loop between IFNγ and pyroptosis in PA-treated Vγ9Vδ2-T cells. This positive loop occurred when PA-induced Vγ9Vδ2-T cells secret excessive IFNγ, leading to pyroptosis of Vγ9Vδ2-T cells, which in turn further stimulated IFNγ secretion from Vγ9Vδ2-T cells. Therefore, inhibition of pyroptosis may serve as an alternative therapeutic strategy to inhibit IFNγ-induced cell pyroptosis. Here, we demonstrated that DMF could rescue the defects of PA-treated Vγ9Vδ2-T cells against tumors by preventing cell pyroptosis. Now it is worth further evaluating the potential of DMF to prevent Vγ9Vδ2-T cell pyroptosis and enhance their antitumor activity in clinical studies. Since DMF is a medication targeting multiple sclerosis,^52^ this novel utilization of an old medication may provide a convenient and secure alternative for enhancing γδ-T cell-mediated antitumor immunity.

While our work has strengths in uncovering the unique functions of PA or OA in Vγ9Vδ2 T-cells against tumor cells, utilizing antibody blockade to specifically assess the role of IFNγ, and employing models of human primary cells that are highly relevant to humans, there are several limitations that should be acknowledged for future research. In our retrospective clinical study, we observed an association between the levels of PA or OA and the efficacy of Vγ9Vδ2-T cell-based anticancer therapy in patients. Although these results provided evidence to the notion that dietary OA could enhance clinical responses to Vγ9Vδ2-T cell-based immunotherapy, the sample size was limited, and a larger cohort study is warranted. We did not explore the effects of blocking or deleting IFNγ in mice models that are immunocompetent, although the Fry revealed that blockade of IFNγ in a leukemia model of immunocompetent mice could not hinder tumor eradication following treatment with wild-type CAR-T cells,^53^ corroborating our results. Furthermore, while our findings demonstrated that IFNγ blockade in Vγ9Vδ2 T-cells enhances their antitumor efficacy, it is important to consider that patients who have not responded well to Vγ9Vδ2-T cell-based immunotherapies and have elevated serum levels of PA may be hesitant to receive IFNγ-blocking antibodies due to the potential risk of reversing the antitumor response and increasing susceptibility to systemic opportunistic infections in advanced cancer patients.^54^ Despite extensive evidence linking IFNγ to tumor immunosurveillance, there are limited success in IFNγ-based therapies in clinical trials.^55^ Our findings may not be directly applicable to cancer patients with metabolic comorbidities, as these conditions could potentially affect the efficacy of dietary interventions. Future research utilizing patient-derived systems and diverse clinical cohorts with varying metabolic profiles is essential to address these gaps and enhance the translational relevance of our findings.

## Materials and methods

### Study design

The aim of this research was to explore how saturated and unsaturated FAs influence the efficacy of γδ-T cells toward cancers and elucidate the underlying mechanisms. Firstly, we determined the level of serum FAs in a group of cancer patients undergoing Vγ9Vδ2-T cell therapy and discovered an association between the levels of PA or OA and the efficacy of Vγ9Vδ2-T cell therapy. Then high PA or OA were used to mimic various conditions that are richer in FAs in human body. We determined the cytotoxic capacity of Vγ9Vδ2-T cells under PA or OA conditions in killing tumor cells and measured the secretions of lytic granules. To explore the mechanism underlying the differential antitumor activity induced by PA and OA, we conducted proteomics analysis of PA- or OA-treated Vγ9Vδ2-T cells and identified IFNγ as a dominant driver of cell pyroptosis, which resulted in the decreased Vγ9Vδ2-T cells’ antitumor activity. We characterized the pathway through western blot analysis and assessed the impact of IFNγ and downstream signalings in the antitumor activity using in vitro cytotoxicity assays, along with blocking antibodies and inhibitor of pyroptosis. Furthermore, we validated the significance of IFNγ in the Vγ9Vδ2-T cells during eliminating tumors using a mouse model.

### Cell culture

Human PBMCs were extracted from the buffy coat of healthy donors, sourced from the Hong Kong Red Cross, using a Ficoll-Paque density gradient method. The study protocols received approval from the Institutional Review Boards of the involved hospitals and universities, ensuring compliance with ethical standards. To prepare the fatty acid solution, fatty acid free-bovine serum albumin (BSA, A7030, sigma) was dissolved in phosphate-buffered saline (PBS) to receive a stock solution with a concentration of 2.5 mM. PA (P0500, Sigma) and OA (O1008, Sigma) were dissolved at a stocking concentration of 15 mM and then combined with BSA at a ratio of 6:1. For Vγ9Vδ2-T cell expansion, PBMCs were treated with PAM (9 µg/ml, 57248-88-1, Hospira, Inc.) and recombinant human IL-2 (200 IU/ml, 11360832, Invitrogen) in RPMI medium (31800022, Gibco) as we described before.^8^ The PBMCs were cultured under four different conditions for approximately 14 days: BSA alone, PA alone, OA alone, or a combination of PA and OA (at a concentration of 50 µM).

The HK-1 nasopharyngeal carcinoma (NPC) cell line was generously provided by Professor S. W. Tsao (The University of Hong Kong), a long-standing collaborator. Human tumor cell lines, including K562 (CCL-243), MCF-7 (HTB-22), HeLa (CCL-2), A549 (CCL-185), and SK-N-BE2 (CRL-2271), were procured from the American Type Culture Collection (ATCC). The A2780 cell line (HTL98008) was obtained from Biovector NTCC Inc. All tumor cell lines were cultured in RPMI medium containing 10% heat-inactivated fetal bovine serum (FBS, 10270-106, Gibco).

### Mice

Rag2^–/–^γc^–/–^ mice aged 6–8 weeks were maintained in the Laboratory Animal Unit at the University of Hong Kong. All experiments involving animals adhered to the regulations established by the University of Hong Kong Committee on the Use of Live Animals in Teaching and Research. The committee formally approved the experimental protocols.

The mice were randomly allocated into four groups. Before tumor implantation, each group was fed one of four specially formulated diets for 1 month: LFD containing 10% fat, a palm oil HFD comprising 45% fat and high in 16:0 PA, an olive oil HFD with 45% fat and rich in 18:1 OA, or a combined palm oil and olive oil HFD, also with 45% fat and enriched with both 16:0 PA and 18:1 OA.

To develop a mouse model of human lung cancer, the mice underwent subcutaneous implantation of the GFP^+^ A549 tumor cell line at a concentration of 0.1 million cells. On the seventh day following inoculation, the mice received intravenous administration of purified Vγ9Vδ2-T cells in a number of 10 million per mouse, which expanded from PBMCs of healthy donors treated with BSA, PA, OA, or PA + OA for 14 days under stimulation with PAM and IL-2 at the specific time points. To investigate how IFNγ influences the capacity of Vγ9Vδ2-T cells in combating tumors, human anti-IFNγ antibody (anti-IFNγ Ab, 12 mg/kg, 506534, Biolegend) or recombinant human IFNγ (rhIFNγ, 8000IU/dose, 300-02, Peprotech) was intraperitoneally injected into the mice 12 h before Vγ9Vδ2-T cell injection. To investigate how pyroptosis influences the capacity of Vγ9Vδ2-T cells in combating tumors, Dimethyl Fumarate (DMF, 50 mg/kg, 242946, Sigma), an inhibitor of pyroptosis, was resolved with 0.5% sodium carboxymethyl cellulose (CMC-Na, HY-Y0703, MedChemExpress) and then intragastrically injected into the mice 12 h before Vγ9Vδ2-T cell injection. Mice receiving an equivalent volume of 0.5% CMC-Na were used as the controls.

Tumor size was assessed at the indicated time after subcutaneous inoculation using whole-body fluorescence imaging with an in vivo imaging instrument with PE IVIS spectrum. Mouse body weight was monitored biweekly. The size of tumors and the survival of the animals were evaluated daily and recorded at the appropriate time. In accordance with the protocols established by the Laboratory Animal Unit at the University of Hong Kong, mice bearing subcutaneous tumors exceeding 17 mm in diameter were euthanized. Tumor size was determined using the formula: length × (width)^2^ × 0.52.

### Patient samples

Serum from hepatocellular carcinoma (HCC) patients who had administrated Vγ9Vδ2-T cell therapy was collected from Zhuhai People’s Hospital (Zhuhai Clinical Medical College of Jinan University), Guang Dong, China. Supplementary Table S1 contains relevant patient information. All clinical studies were authorized by the ethics committee of Zhuhai People’s Hospital, and an informed consent form was signed by each enrolled patient. The survival time was determined by calculating the duration from the time of the patient enrollment until October 2023.

### In vitro cytotoxicity assay

After a 14-day treatment under FA treatment, Vγ9Vδ2-T cells, referred to as effector cells (E), were isolated by γδ-T cell negative selection (130-092-892, Miltenyi Biotec). Subsequently, the purified Vγ9Vδ2-T cells were cocultured with tumor cells, referred to as target cells (T), for 6 h (E/T ratio:10:1).^56^

To study how IFNγ affects the Vγ9Vδ2-T cells’ antitumor function, 3 days prior to the co-culture with tumor cells, IFNγ-mediated pathways were activated with rhIFNγ at a dose of 100 ng/ml and blocked with anti-IFNγ Ab (10 µg/ml). Subsequently, anti-TCR Vδ2 and anti-CD3 antibodies were utilized to stain Vγ9Vδ2-T cells. Annexin V/Propidium Iodide (PI) (640914, Biolegend) staining was utilized to distinguish apoptotic cells. As previously reported, flow cytometry was used to measure the cytotoxic ability of Vγ9Vδ2-T cells in killing tumor cells by calculating the proportion of Annexin V^+^ cells in the CD3^-^ population.^21^

To explore how PA affects pyroptosis in Vγ9Vδ2-T cells, DMF was utilized to treat Vγ9Vδ2-T cells under BSA or PA conditions. Moreover, nigericin sodium salt (HY-100381, MedChemExpress), the inducer of pyroptosis, was used to treat Vγ9Vδ2-T cells under BSA or OA conditions. Similarly, to investigate how IFNγ affects the pyroptosis pathway in Vγ9Vδ2-T cells, DMF was utilized to treat Vγ9Vδ2-T cells under BSA or OA conditions, with or without the presence of rhIFNγ. Next, K562 tumor cells were cultured with the treated Vγ9Vδ2-T cells, and flow cytometry was used to examine the apoptotic cells.

To determine how IFNγ affects pyroptosis in Vγ9Vδ2-T cells, fludarabine (HY-B0069, MedChemExpress) and 1400 W (HY-18731, MedChemExpress), the inhibitors of STAT1 phosphorylation and NO synthesis, respectively, were utilized to treat Vγ9Vδ2-T cells under BSA or PA conditions. Similarly, to investigate how IFNγ affects the pyroptosis pathway of Vγ9Vδ2-T cells, fludarabine or 1400 W were cultured with Vγ9Vδ2-T cells under BSA or OA conditions, with or without the presence of rhIFNγ. Then, after coculturing treated Vγ9Vδ2-T cells with K562 tumor cells, apoptotic tumor cells were performed analysis using flow cytometry.

### Cell apoptosis and proliferation assay

After a 14-day treatment under FA treatment, anti-TCR Vδ2 and anti-CD3 antibodies were used to stain Vγ9Vδ2-T cells and Annexin V/PI was utilized to distinguish apoptotic cells by flow cytometry.

To investigate proliferation, CD3 T cells were labeled for Carboxyfluorescein succinimidyl ester (CFSE, 21888, Sigma) as previously reported.^21^ CFSE fluorescence levels were measured by flow cytometry.

### Flow cytometric analysis

For surface marker staining, the antibodies below were utilized: anti-TCR Vδ2 (331418, Biolegend), anti-CD3 (300330, Biolegend), anti-CD36 (336230, Biolegend), anti-CD4 (300524, Biolegend), anti-CD8 (980918, Biolegend), and Fixable Viability Dye (65-0866-14, Thermo Fisher Scientific). For the phosphorylated antibody and transcription factor analysis, cells were fixed, permeabilized and stained with anti-STAT1 phospho (Tyr701) antibody (666404, Biolegend) or anti-IRF1 (332804, Biolegend). For the intracellular staining, cells were fixed, permeabilized and subsequently incubated with anti-iNOS (MA5-17139, Thermo Fisher Scientific) and then staining with secondary antibody (A-11001, Thermo Fisher Scientific). Flow cytometry was used to assess cellular phenotypes. Then the data analysis was analyzed using FlowJo v10 as we described previously.^57,58^

### FlowCytomix assay

After coculturing Vγ9Vδ2-T cells with tumor cells for 6 h, the supernatant was collected. A human CD8/NK panel (13-plex) kit (740267, Biolegend) was used to measure the secretions of lytic granules. Flow cytometry was used to calculate the concentration of cytotoxic cytokines. The results were performed analysis through LEGENDplex^TM^ Data Analysis software (Biolegend).

### LipidTox analysis

After a 14-day treatment under BSA, PA, OA, or a combination of PA and OA conditions, Vγ9Vδ2-T cells were incubated with LipidTOX™ Green Neutral Lipid (H34475, Thermo Fisher Scientific) for 30 min at 37 °C. The data was analyzed using flow cytometry and FlowJo v10.

After treatment with BSA, PA, OA, or PA + OA, purified Vγ9Vδ2-T cells were pretreated with LipidTOX™ Red Phospholipidosis Detection Reagent (H34351, Thermo Fisher Scientific) in 37 °C incubator for 48 h. Then the cells were collected, fixed, washed, and incubated for 30 min with LipidTOX™ Green Neutral Lipid (H34475, Thermo Fisher Scientific). Finally, the cells were attached to a slide and mounted. The lipid staining was assessed using a Zeiss LSM 980. ZEN (version 2.3) software was used to perform image analysis.

### Intracellular nitric oxide (NO) measurement

A NO fluorescence probe (DAF-FM DA, S0019S, Beyotime Biotechnology) was used to determine the intracellular NO release by a fluorescent microscope. Briefly, Vγ9Vδ2-T cells were incubated in pre-warmed PBS containing the DAF-FM DA probe with a final concentration of 1 µM, and anti-TCR Vδ2 (331418, Biolegend) and then incubated for 30 min at 37 °C. Then the cells were washed, fixed, adhered to the slide, and mounted with ProLongTM Mountant with DAPI. The fluorescence images were received by a Zeiss LSM 980. ZEN (version 2.3) software was used to perform image analysis.

### Intracellular ROS and lipid ROS measurement

Vγ9Vδ2-T cells were incubated with pre-warmed PBS containing the C11 BODIPY 581/591 probe (D3861, Thermo Fisher Scientific) or H_2_DCF-DA probe (D399, Thermo Fisher Scientific) with a final concentration of 10 µM, anti-CD3, anti-TCR Vδ2, and Fixable Viability Dye and then incubated for 30 min at 37 °C. CD3 T cells were resuspended in PBS containing the H_2_DCF-DA probe or C11 BODIPY 581/591 probe with a final concentration of 10 µM, anti-CD3, anti-CD4, anti-CD8 and Fixable Viability Dye at 37 °C for 30 min. Then the data was analyzed by flow cytometry.

### Real-time quantitative polymerase chain reaction (RT-qPCR) and immunoblotting

For RNA analysis and immunoblotting analysis, the protocol was described previously.^21^ The primers used were purchased from Sangon Biotech. The primary antibodies include anti-GSDMD (97558, Cell Signaling Technology), anti-cleaved GSDMD (36425, Cell Signaling Technology), anti-caspase 1 (3866, Cell Signaling Technology), anti-cleaved caspase 1 (4199, Cell Signaling Technology), or anti-GAPDH (2118, Cell Signaling Technology).

### ELISA

The supernatant of purified Vγ9Vδ2-T cells after being treated as indicated was collected and used to determine IFNα and IFNγ level using human IFNα (E-EL-H6125, Elabscience) and IFNγ ELISA kit (E-EL-H0108c, Elabscience) according to the standard protocol. The serum collected from Rag2^−/−^γc^−/−^ mice was used to examine the level of adiponectin (EK295, MultiSciences), insulin (E-EL-M1382c, Elabscience), leptin (EK297, MultiSciences), and resistin (EK2R01, MultiSciences) based on the standard protocol. The absorbance was detected by BMG CLARIOstar Plus and the level of these cytokines was calculated using the standard curve.

### LDH release detection

The supernatant from purified Vγ9Vδ2-T cells after being treated as indicated was collected and used to detect LDH level using LDH-Glo™ Cytotoxicity Assay (J2380, Promega) according to the standard protocol. The luminescence signals were measured by BMG CLARIOstar Plus and the level of LDH release was calculated using the standard curve.

### NO release detection

The levels of NO in the supernatant of purified Vγ9Vδ2-T cells, treated as specified, were quantified using the CheKine^TM^ Micro Nitric Oxide Assay Kit (KTB1400, Abbkine) following the manufacturer’s protocol.

### Glycolysis and OXPHO analysis

ECAR and OCR in purified Vγ9Vδ2-T cells treated as indicated were analyzed by a Seahorse XF96 extracellular Flux Analyzer (Agilent) as we described previously.^21^

### Proteomics analysis

Proteins were extracted from purified Vγ9Vδ2-T cells, and the protein concentrations were then quantified by Bradford, and the quality control was conducted by SDS-PAGE. Protein from each group was digested with the trypsin enzyme. Then, A Strata X column was then used to desalt the enzymatic peptides, which were separated using HPLC. DDA library construction and DIA quantification were conducted by nano-LC-MS/MS. DDA data were analyzed by Andromeda search engine integrated within MaxQuant, with identification outcomes employed to construct a spectral library. Quantitative analysis revealed differentially expressed proteins across comparison groups. Subsequently, functional pathway enrichment analysis was conducted using the Enrichr database (https://maayanlab.cloud/Enrichr/).

### Targeted metabolism for FAs

Serum was collected from HCC patients with γδ-T cell therapy. The metabolomics was performed to quantitatively measure 48 free FAs using a GC-EI-MS/MS system, comprising A 7000D mass spectrometer connected to an Agilent 7890B gas chromatograph. The Metware Database (MWDB), created by Wuhan Metware Biotechnology Co. (Wuhan, China), was utilized for data analysis.

### Statistics

Quantitative data were presented as mean ± standard error of the mean (SEM). One-way analysis of variance (ANOVA) followed by Tukey’s correction was employed for multiple group comparisons. Serum FA levels in patients were compared using two-way ANOVA. The Pearson correlation test was utilized to assess relationships between variables. Tumor volume and mice survival were evaluated using two-way ANOVA and the Kaplan-Meier log-rank test, respectively. Data was significant when *p* < 0.05. Specific sample size and *p*-value were described in the figure legends.

## Supplementary information

supplemental materials

## Data Availability

The data supporting this article’s findings are available from the corresponding author upon reasonable request. Proteomics data are available via ProteomeXchange with identifier PXD064016.
