# Supplementary material for: Oleic acid restores the impaired antitumor immunity of γδ-T cells induced by palmitic acid
Source: Signal Transduct Target Ther. 2025 Jul 3;10:209. doi: 10.1038/s41392-025-02295-8 (PMC12222472; doi:10.1038/s41392-025-02295-8)

Supplementary Materials for

**Oleic acid restores the impaired antitumor immunity of γδ-T cells induced by**

**palmitic acid**

Yanmei Zhang, Zheng Xiang, Yan Xu, Lo Sha Cheung, Xiwei Wang, Manni Wang, Howard Ho Wai Wong, Zhenyao Zhu, Wenyue Zhang, Yifan Gao, Xianze Luo, Yin Celeste Cheuk, Yixin Zhou, Xianfeng Zha, Yashi Chen, Man Li, Feifei Luo, Yiwei Chu, Yu-Lung Lau, Yinping Liu, and Wenwei Tu*

Correspondence to: [wwtu@hku.hk](mailto:wwtu@hku.hk)

**This PDF file includes:**

Figures. S1 to S9

Tables S1

Original and uncropped films of Western blots

Gating strategies for flow cytometry


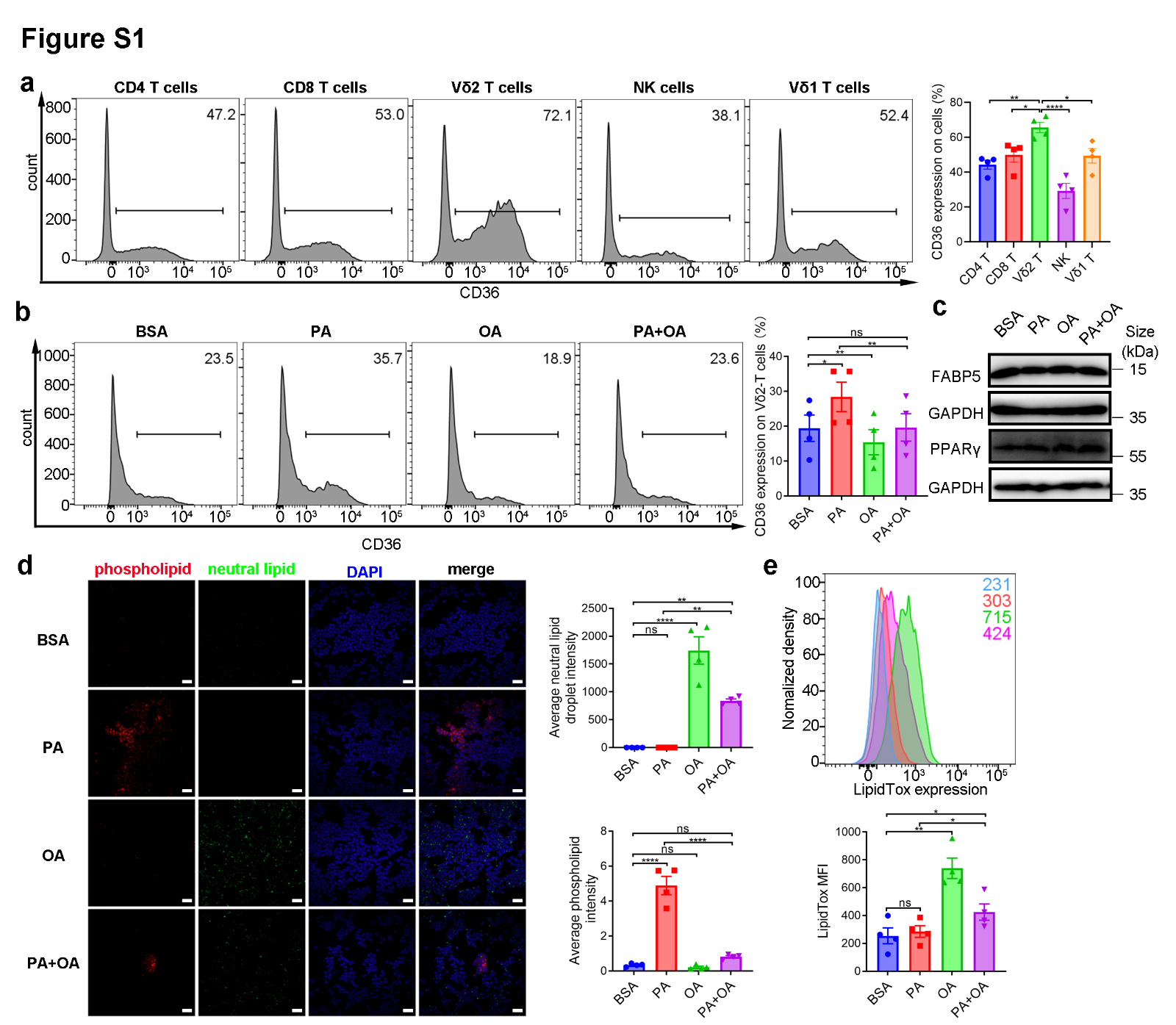


**Supplementary Fig. 1. PA increases while OA decreases CD36 expression on Vγ9Vδ2-T cells.** **a.** CD36 expression on the CD4 T cells, CD8 T cells, Vγ9Vδ2-T cells, NK cells, or Vγ9Vδ1-T cells from PBMC of healthy donors. Representative images and quantification of CD36 expression are shown (n = 4). **b.** Vγ9Vδ2-T cells were cultured with BSA, PA, OA, or a mixture of PA and OA for 5 days, and then CD36 expression was detected by flow cytometry. Representative images and quantification of CD36 expression are shown (n = 4). **c.** Vγ9Vδ2-T cells were expanded from PBMCs and cultured with BSA, PA, OA, or a mixture of PA and OA for 14 days. The expression of FABP5 and PPARγ in purified Vγ9Vδ2-T cells were examined by western blot. **d.** Vγ9Vδ2-T cells were cultured with BSA, PA, OA, or a mixture of PA and OA for 14 days, then stained with phospholipid (red), neutral lipid (green), and DAPI (blue). Representative confocal images and quantification of average lipid intensity are shown (n = 4). The scale bar represents 10 µm. **e.** Lipids level of Vγ9Vδ2-T cells was measured by lipidTox staining by flow cytometry. Representative images and quantification of lipidTox MFI are shown (n = 4). The data are shown as the mean ± SEM. *ns*, not significant; **p* < 0.05; ***p*<0.01; *****p* < 0.0001.


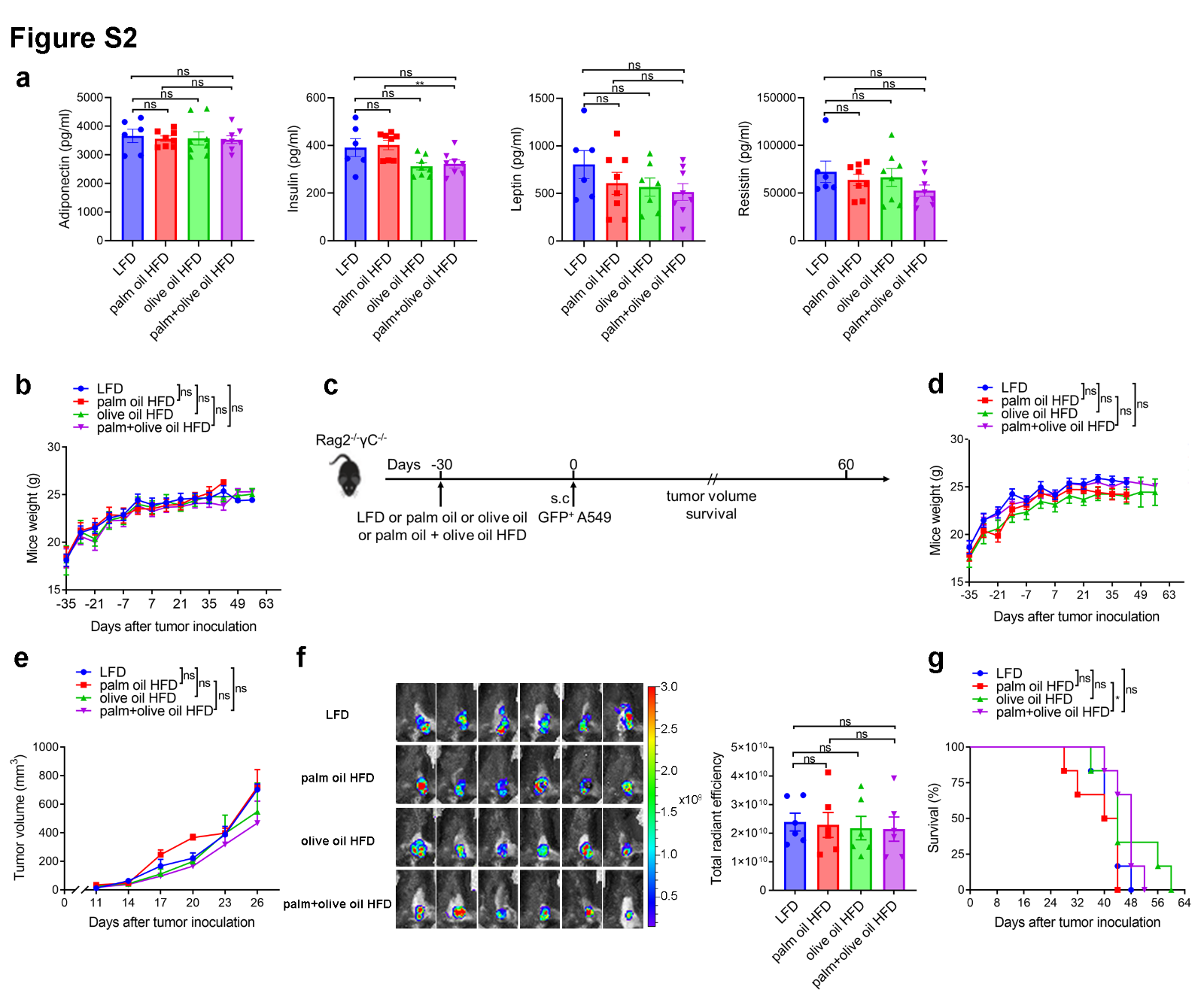


**Supplementary Fig. 2. Dietary high in palm oil or olive oil has no significant impact on tumor progression in Rag2^−/−^γc^−/−^ mice. a** and **b.** Rag2^−/−^γc^−/−^ mice fed on the LFD, palm oil, olive oil or palm and olive oil HFDs for 30 days were subcutaneously (s.c.) injected with GFP^+^ A549 tumor cells (0.1 × 10^6^ cells per mouse). Expanded BSA-, PA-, OA- or PA+OA-Vγ9Vδ2-T cells (10 × 10^6^ cells per mouse) were intravenously (i.v.) transferred into the mice at the indicated times. **a.** Analysis of systemic metabolic parameters, including adiponectin, insulin, leptin, and resistin in Rag2^−/−^γc^−/−^ mice fed with LFD, palm oil, olive oil or palm and olive oil HFDs for 30 days. **b.** Mouse body weight of Rag2^−/−^γc^−/−^ mice fed on LFD, palm oil, olive oil or palm and olive oil HFDs for 2 months (n = 6 mice per group). **c.** Diagram of the experimental paradigm in **d-g**. Rag2^−/−^γc^−/−^ mice fed on the LFD, palm oil, olive oil or palm and olive oil HFDs for 30 days were subcutaneously (s.c.) injected with GFP^+^ A549 tumor cells (0.1 × 10^6^ cells per mouse) (n = 6 mice per group). **d.** Mouse body weight of Rag2^−/−^γc^−/−^ mice fed on LFD, palm oil, olive oil or palm and olive oil HFDs for 2 months. **e.** Tumor volumes were obtained at the indicated times. **f**. Whole-body fluorescence images (left) and total radiant efficiency of fluorescence intensity (right) of the mice were assessed on day 26 after the inoculation of GFP^+^ A549 tumor cells. **g.** Survival curves were obtained at the indicated times. Quantitative data are shown as the mean ± SEM. *ns*, not significant; **p* < 0.05; ***p*<0.01; *****p* < 0.0001.


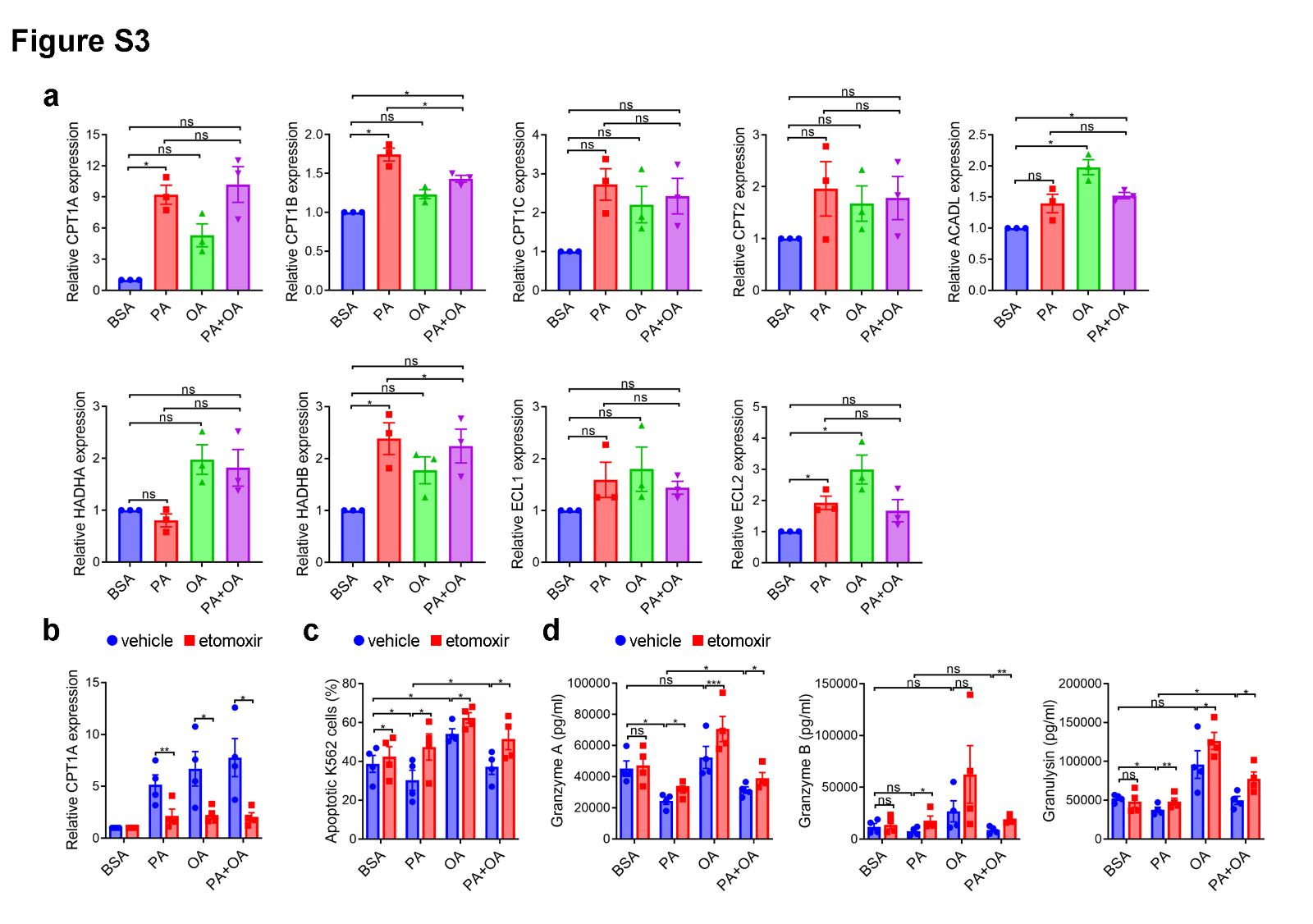


**Supplementary Fig. 3. Blockade of CPT1 rescues the impaired antitumor activity of Vγ9Vδ2-T cells induced by PA.** **a.** RT-qPCR analysis of CPT1A, CPT1B, CPT1C, CPT2, ACADL, HADHA, HADHB, ECL1, and ECL2 gene expression in purified Vγ9Vδ2-T cells treated with BSA alone, 50 µM PA, 50 µM OA, or a combination of 50 µM PA and OA for 14 days was performed (n = 3). **b-d.** Vγ9Vδ2-T cells were cultured with BSA alone, 50 µM PA, 50 µM OA, or a combination of 50 µM PA and OA for 14 days, then treated with or without CPT1 inhibitor etomoxir for 24 hours. **b.** RT-qPCR analysis of CPT1A in Vγ9Vδ2 T cells was determined (n = 4). **c.** The anti-tumor activity of expanded BSA-, PA-, OA- or PA+OA-Vγ9Vδ2-T cells was detected by co-culturing Vγ9Vδ2-T cells with K562 tumor cells at an effector/target cells of 10:1 for 6 hours, and then the apoptotic K562 cells were determined by flow cytometry (n = 4). **d.** The secretions of granzyme A, granzyme B, and granulysin collected from the supernatant of the co-culturing system between BSA, PA, OA, or PA+OA treated Vγ9Vδ2-T cells and K562 tumor cells were measured (n = 4). The data are shown as the mean ± SEM. *ns*, not significant; **p* < 0.05; ***p*<0.01; ****p* < 0.001.


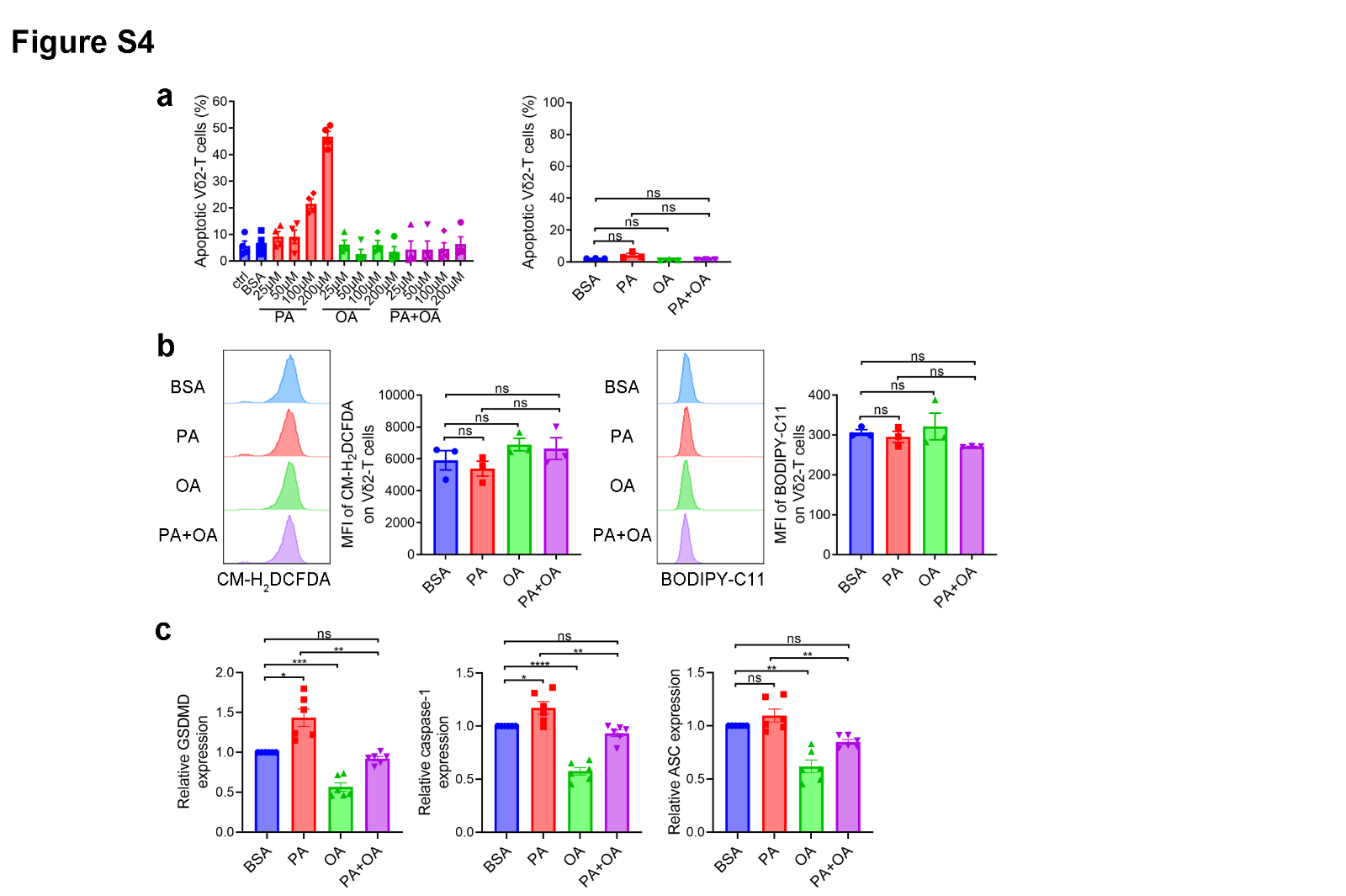


**Supplementary Fig. 4. PA induces pyroptosis of Vγ9Vδ2-T cells, but not apoptosis and ferroptosis. a-c.** Vγ9Vδ2-T cells were cultured with BSA, PA, OA, or a mixture of PA and OA for 14 days. **a.** The apoptosis of Vγ9Vδ2 T cells was examined by flow cytometry (n = 4). **b.** The level of ROS and lipid ROS in Vγ9Vδ2 T cells after 14 days of culture was measured by flow cytometry (n = 3). **c.** RT-qPCR analysis of GSDMD, caspase-1 and ASC in BSA-, PA-, OA-, or PA+OA-Vγ9Vδ2 T cells (n = 4). The data are shown as the mean ± SEM. *ns*, not significant; **p* < 0.05; ***p*<0.01; ****p* < 0.001; *****p* < 0.0001.


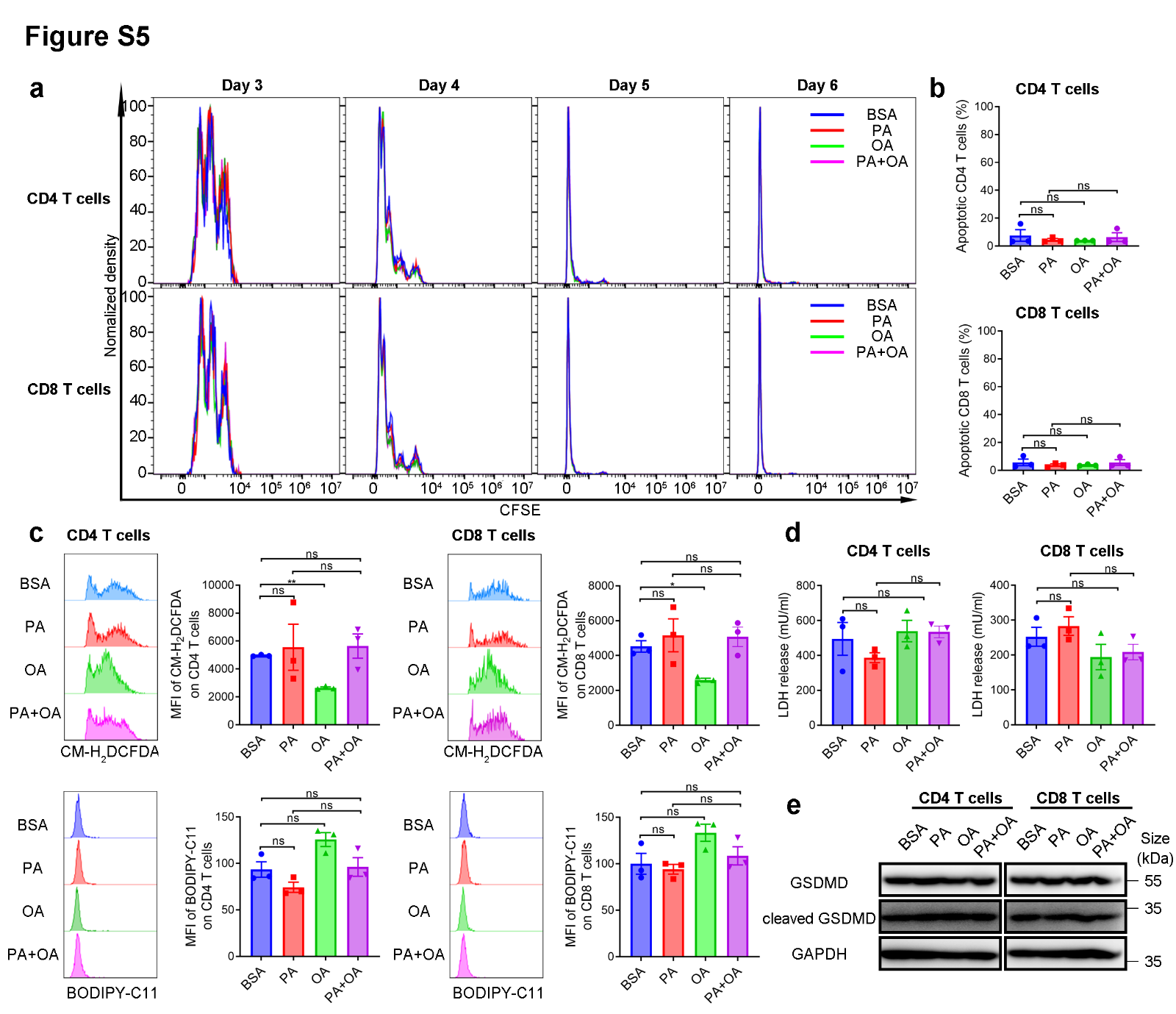


**Supplementary Fig. 5. PA does not induce apoptosis, ferroptosis, and pyroptosis in CD4 or CD8 T cells. a.** Representative graphs of the proliferative response of CD3/CD28 antibody activated CD4 and CD8 T cells were determined by CFSE staining on day 3, 4, 5 and 6. **b-e.** CD3/CD28 antibody activated CD4 and CD8 T cells were cultured with BSA, PA, OA, or a mixture of PA and OA for 14 days. **b.** The apoptosis of CD4 and CD8 T cells was examined by flow cytometry (n = 3). **c.** The level of ROS and lipid ROS in CD4 and CD8 T cells after 14 days of culture was measured by flow cytometry (n = 3). **d.** The level of LDH release in CD3/CD28 antibody activated CD4 and CD8 T cells was examined. **e.** Representative immunoblot of GSDMD, and cleaved GSDMD in BSA-, PA-, OA-, or PA+OA-CD4 T cells (left) and -CD8 T cells (right). GAPDH was used as a loading control. The data are shown as the mean ± SEM. *ns*, not significant; **p* < 0.05; ***p*<0.01.


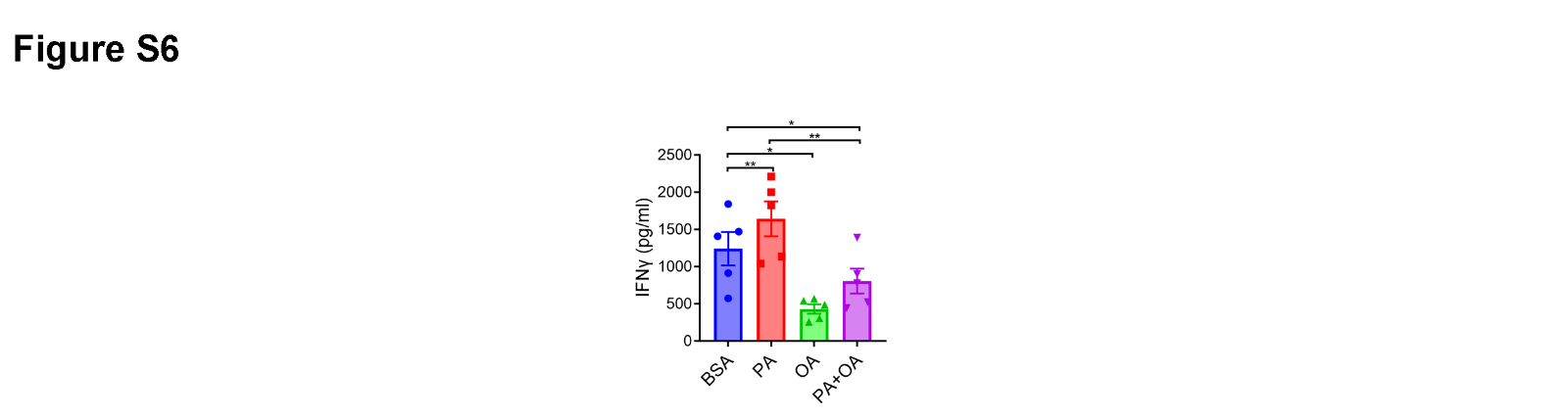


**Supplementary Fig. 6. PA increases while OA decreases the secretion of IFNγ in the cocultured system.** The secretion of IFNγ from the supernatant of coculture BSA-, PA-, OA-, or PA+OA-Vγ9Vδ2-T cells and tumor cells was measured by ELISA (n = 5). The data are shown as the mean ± SEM. *ns*, not significant; **p* < 0.05; ***p*<0.01.


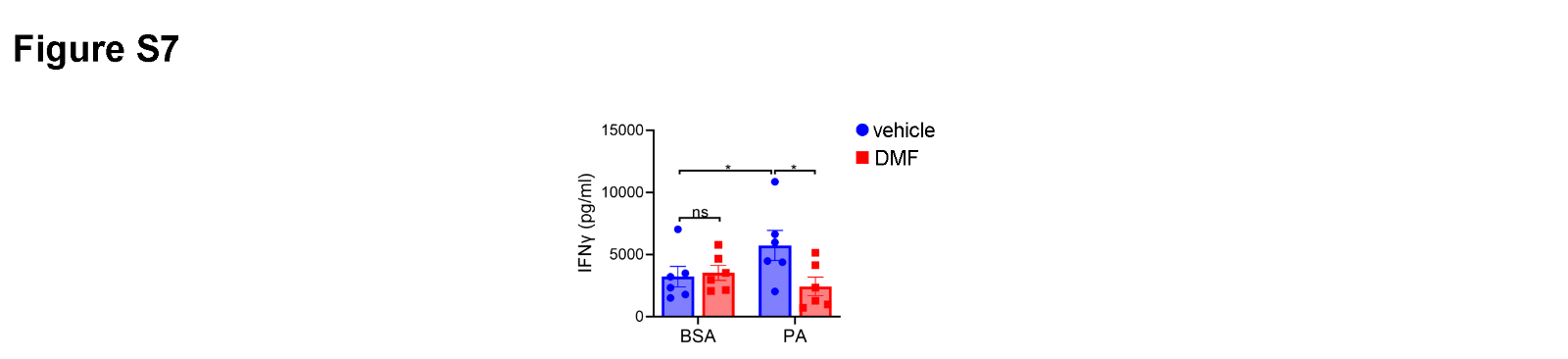


**Supplementary Fig. 7. Inhibition of pyroptosis decreases the secretion of IFNγ from Vγ9Vδ2-T cells.** Expanded BSA-, or PA-Vγ9Vδ2-T cells were treated with DMF to block pyroptosis. The secretion of IFNγ collected from the supernatant of BSA-, or PA-Vγ9Vδ2 T cells was examined (n = 6). The data are shown as the mean ± SEM. *ns*, not significant; **p*<0.05.


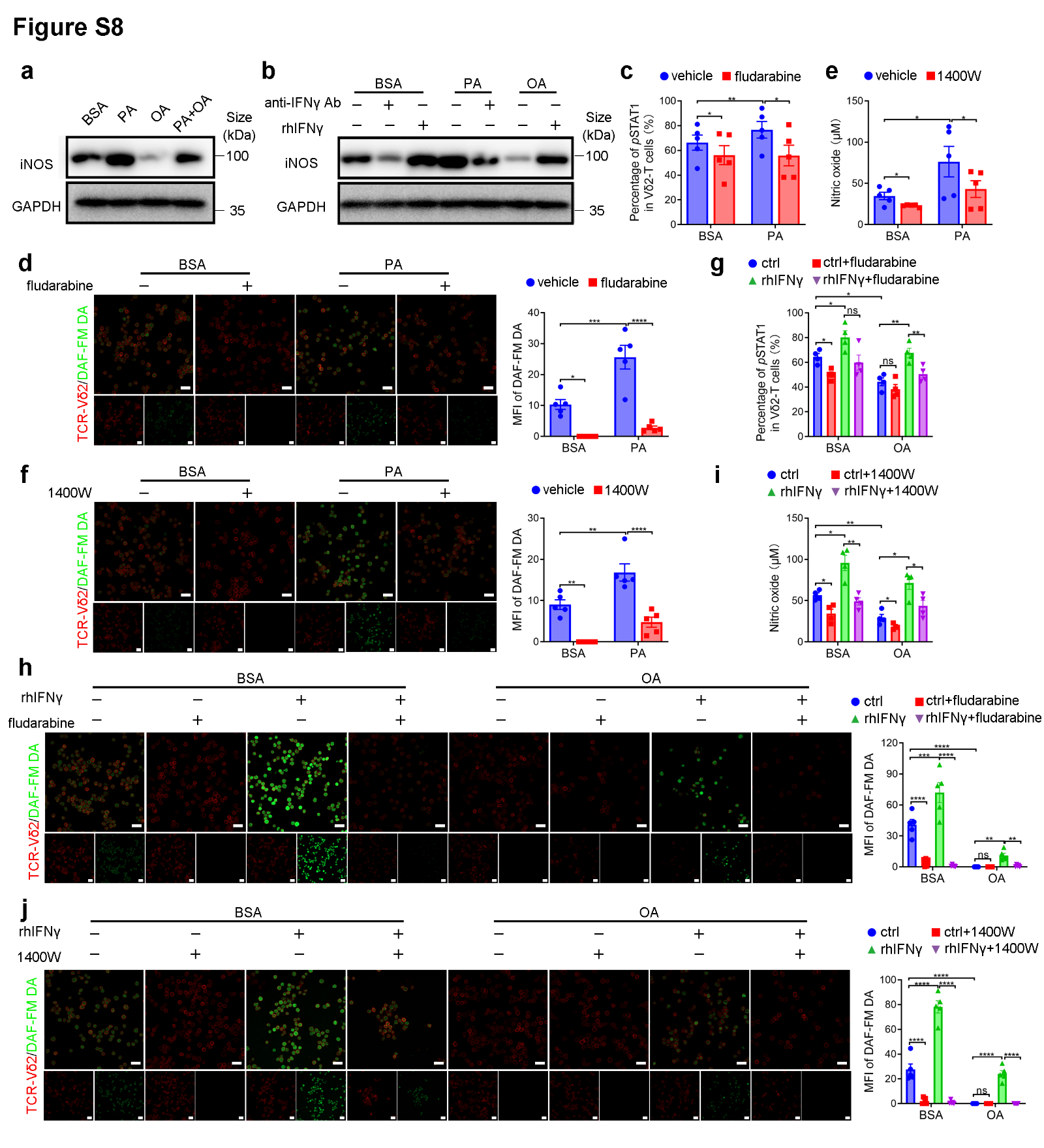


**Supplementary Fig. 8. *p*STAT1-IRF1-iNOS pathway mediates IFNγ-induced pyroptosis in Vγ9Vδ2-T cells. a.** Vγ9Vδ2-T cells were cultured with BSA, PA, OA, or a mixture of PA and OA for 14 days. The expression of iNOS on Vγ9Vδ2-T cells was examined by western blot. **b.** Expanded BSA-, PA- or OA-Vγ9Vδ2-T cells were treated with or without anti-IFNγ Ab or rhIFNγ. The expression of iNOS on Vγ9Vδ2-T cells was examined by western blot. **c-e.** Expanded BSA-, or PA-Vγ9Vδ2-T cells were treated with or without fludarabine or 1400W. **c.** The expression of *p*STAT1 on Vγ9Vδ2-T cells was examined by flow cytometry (n = 5). **d** and **f.** The NO level in Vγ9Vδ2-T cells was quantified using an DAF-FM DA probe under confocal microscopy. Representative confocal images and quantification of average fluorescence intensity are shown (n = 5). The scale bar represents 20 µm. **e.** The level of NO in their supernatants was examined (n = 5). **g-j.** Expanded BSA-, or OA- Vγ9Vδ2-T cells were treated with fludarabine or 1400W, and then rhIFNγ was used. **g.** The expression of *p*STAT1 on Vγ9Vδ2-T cells was examined by flow cytometry (n = 4). **h** and **j.** The NO level in Vγ9Vδ2-T cells was quantified using an DAF-FM DA probe under confocal microscopy. Representative confocal images and quantification of average fluorescence intensity are shown (n = 5). The scale bar represents 20 µm. **i.** The level of NO in their supernatants was examined (n = 4). The data are shown as the mean ± SEM. *ns*, not significant; **p* < 0.05; ***p*<0.01; ****p* < 0.001; *****p* < 0.0001.


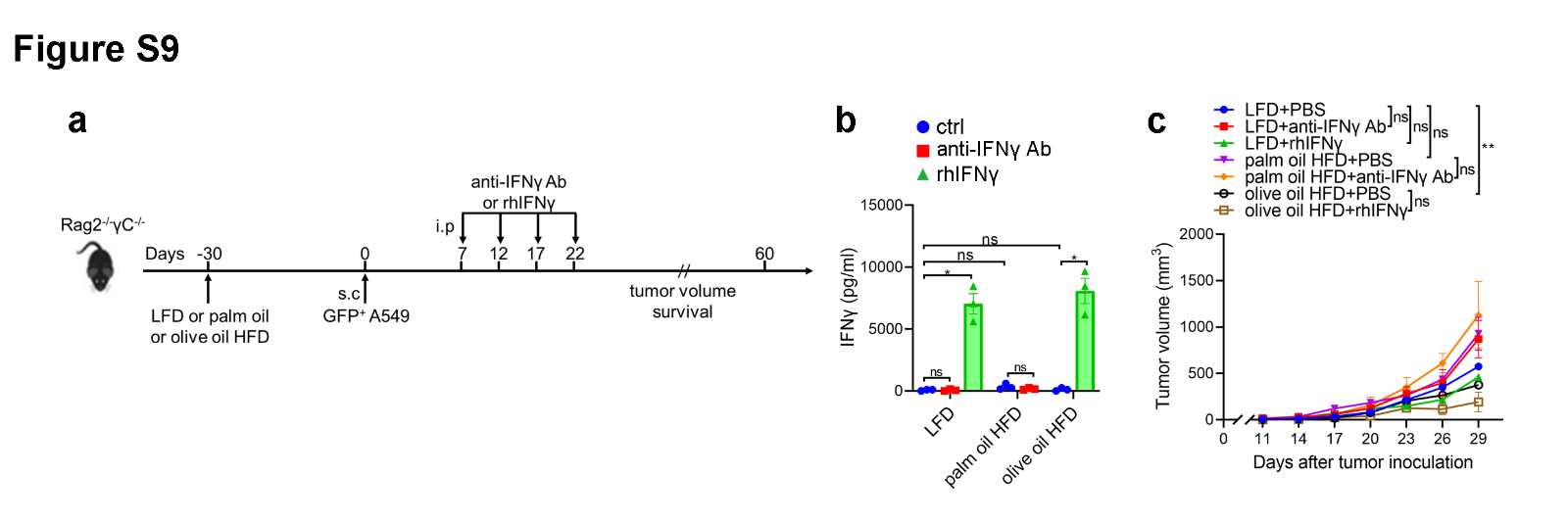


**Supplementary Fig. 9. IFNγ has no significant impact on tumor progression in Rag2^−/−^γc^−/−^ mice. a.** Diagram of the experimental paradigm in **b and c**. Rag2^−/−^γc^−/−^ mice fed on the LFD, palm oil, or olive oil HFDs for 30 days were subcutaneously (s.c.) injected with GFP^+^ A549 tumor cells (0.1 × 10^6^ cells per mouse) (n = 3 mice per group). Then anti-IFNγ Ab or rhIFNγ was intraperitoneally (i.p.) injected into mice. **b.** The level of IFNγ from mice serum collected on the 2^nd^ day after injection of anti-IFNγ Ab or rhIFNγ was measured. **c.** Tumor volumes were obtained at the indicated times. Quantitative data are shown as the mean ± SEM. *ns*, not significant; **p* < 0.05; ***p*<0.01.

| **Patients no** | **Sex** | **Age(year)** | **Weight(kg)** | **Height(m)** | **TG** | **TC** | **HDL-C** | **LDL-C** | **Diagnosis/**  **phase** | **Times of γδ-T cells treatment** | **Clinical**  **response** | **Survive**  **(Yes/No)** | **Survival time**  **(month)** |
| --- | --- | --- | --- | --- | --- | --- | --- | --- | --- | --- | --- | --- | --- |
| 1 | male | 66 | 59 | 1.66 | normal | normal | normal | normal | IIIb | 18 | CR | Yes | >14 |
| 2 | male | 59 | 60 | 1.64 | normal | normal | normal | normal | IIb | 18 | CR | Yes | >13 |
| 3 | male | 52 | 69 | 1.80 | normal | normal | normal | normal | IV | 18 | CR | Yes | >13 |
| 4 | male | 53 | 70 | 1.68 | normal | normal | normal | normal | IIIb | 20 | PA | No | 10 |
| 5 | male | 68 | 60.5 | 1.62 | normal | normal | normal | normal | IIIb | 8 | PA | No | 4 |
| 6 | male | 34 | 62 | 1.63 | normal | normal | normal | normal | IIb | 7 | PA | No | 4 |
| 7 | male | 55 | 64 | 1.60 | normal | normal | normal | normal | IV | 3 | PA | No | 1 |

**Table S1. Background of HCC patients treated with allogeneic γδ-T cells.**

TG: triglyceride; TC: total cholesterol; HDL-C: high-density lipoprotein cholesterol; LDL-C: low-density; CR: complete response; PA: pass away.

**Original and uncropped films of Western blots**


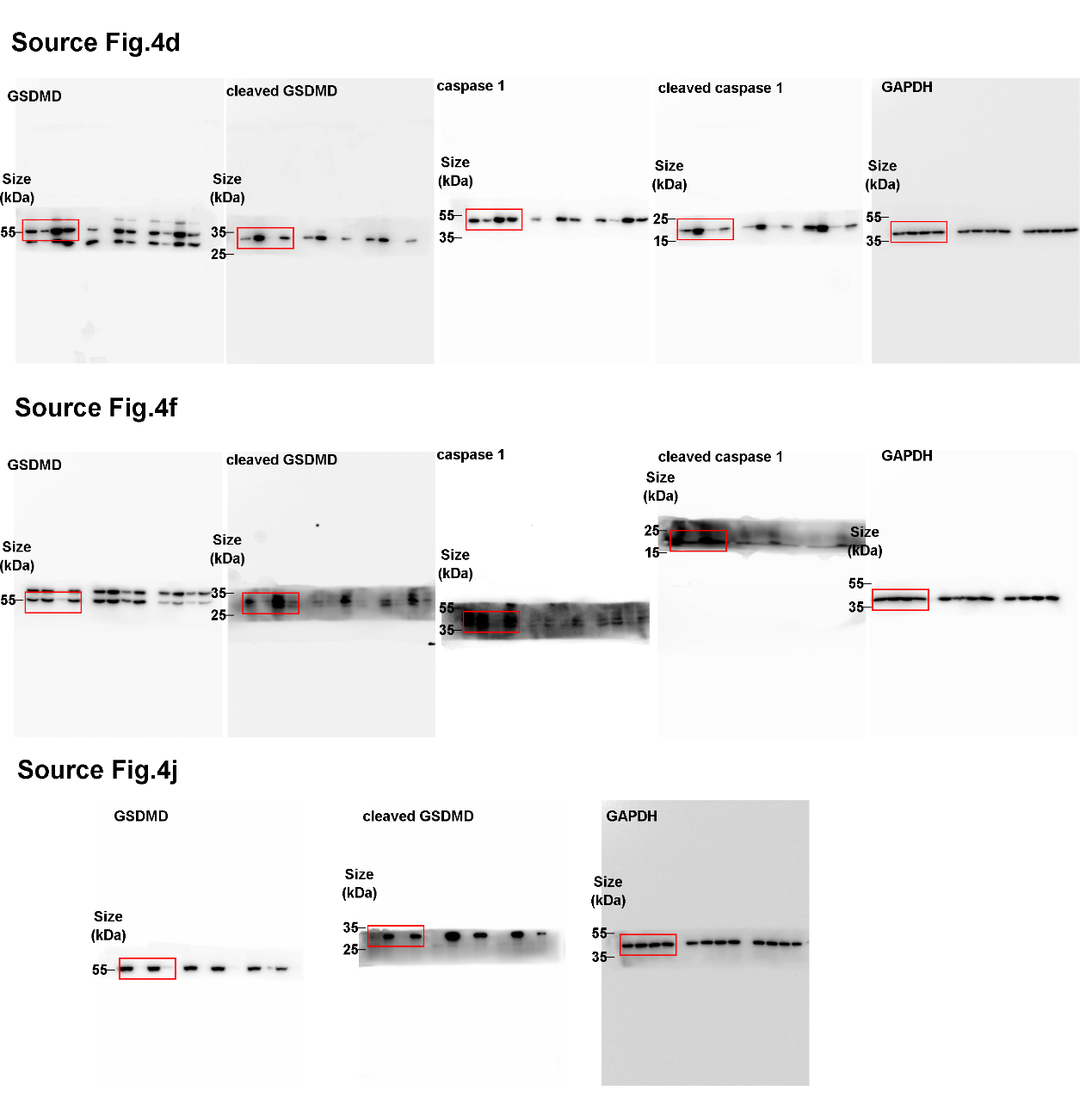


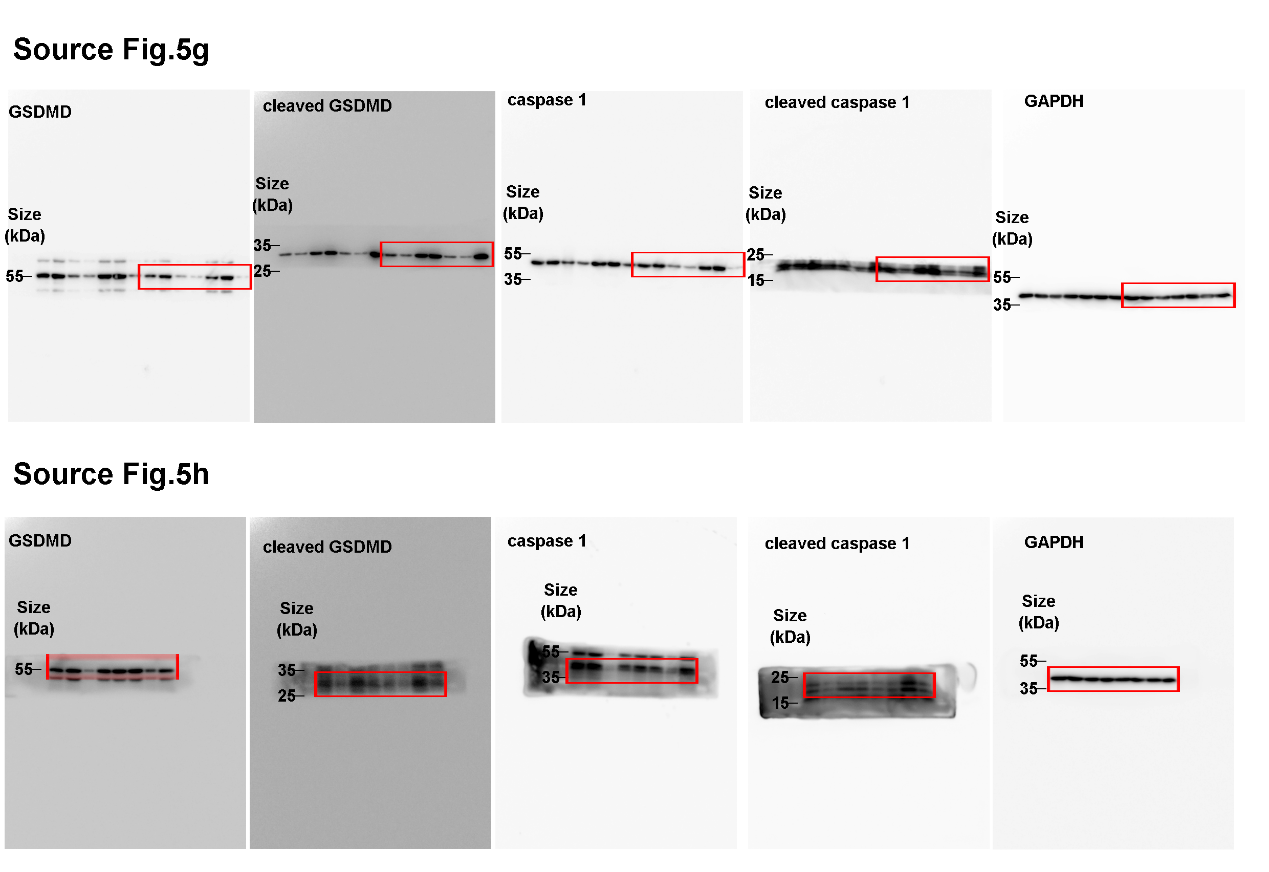


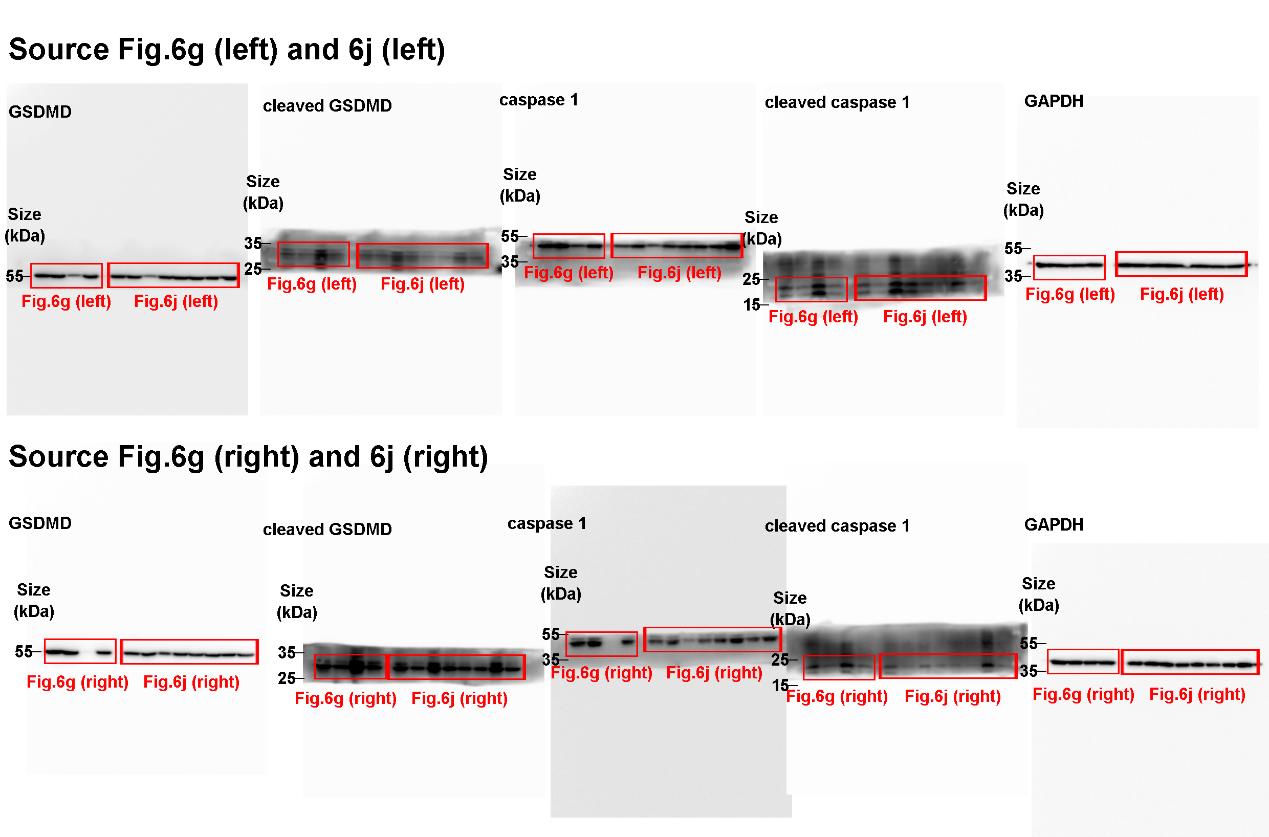


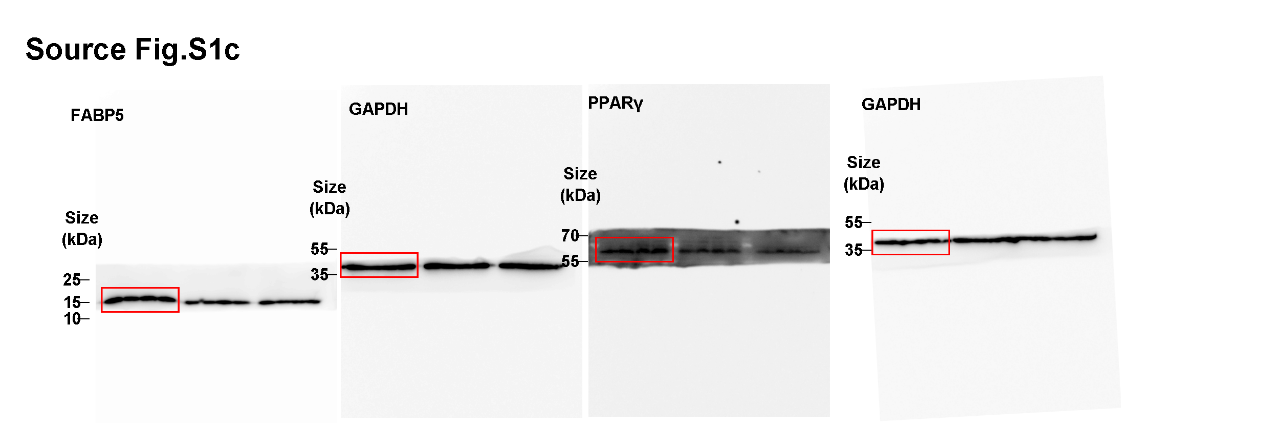


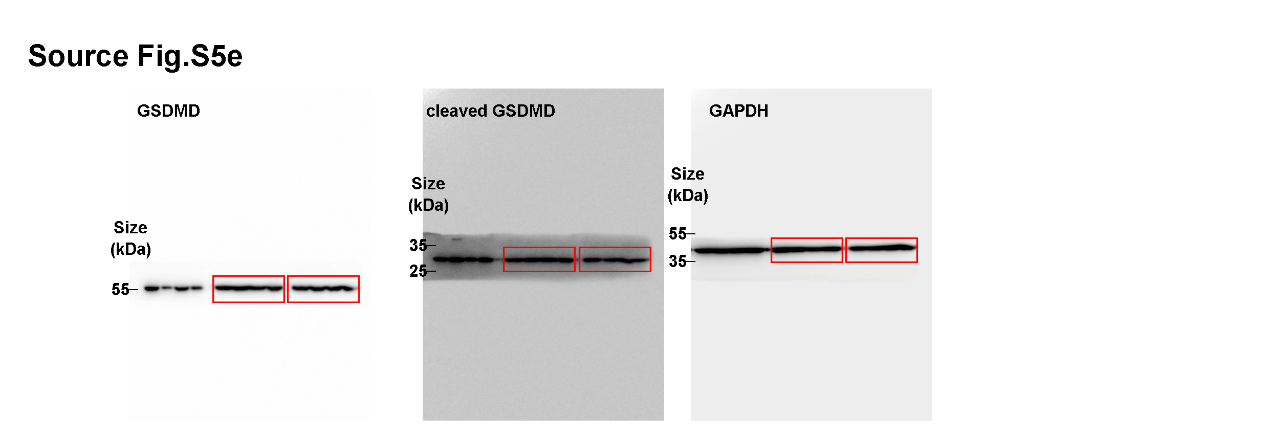


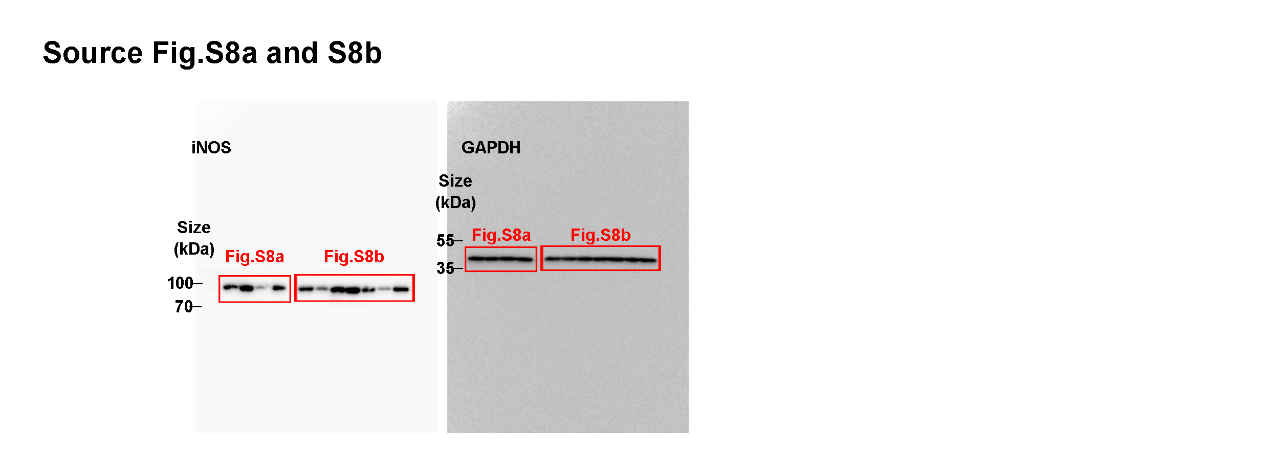


**Gating strategies for flow cytometry**


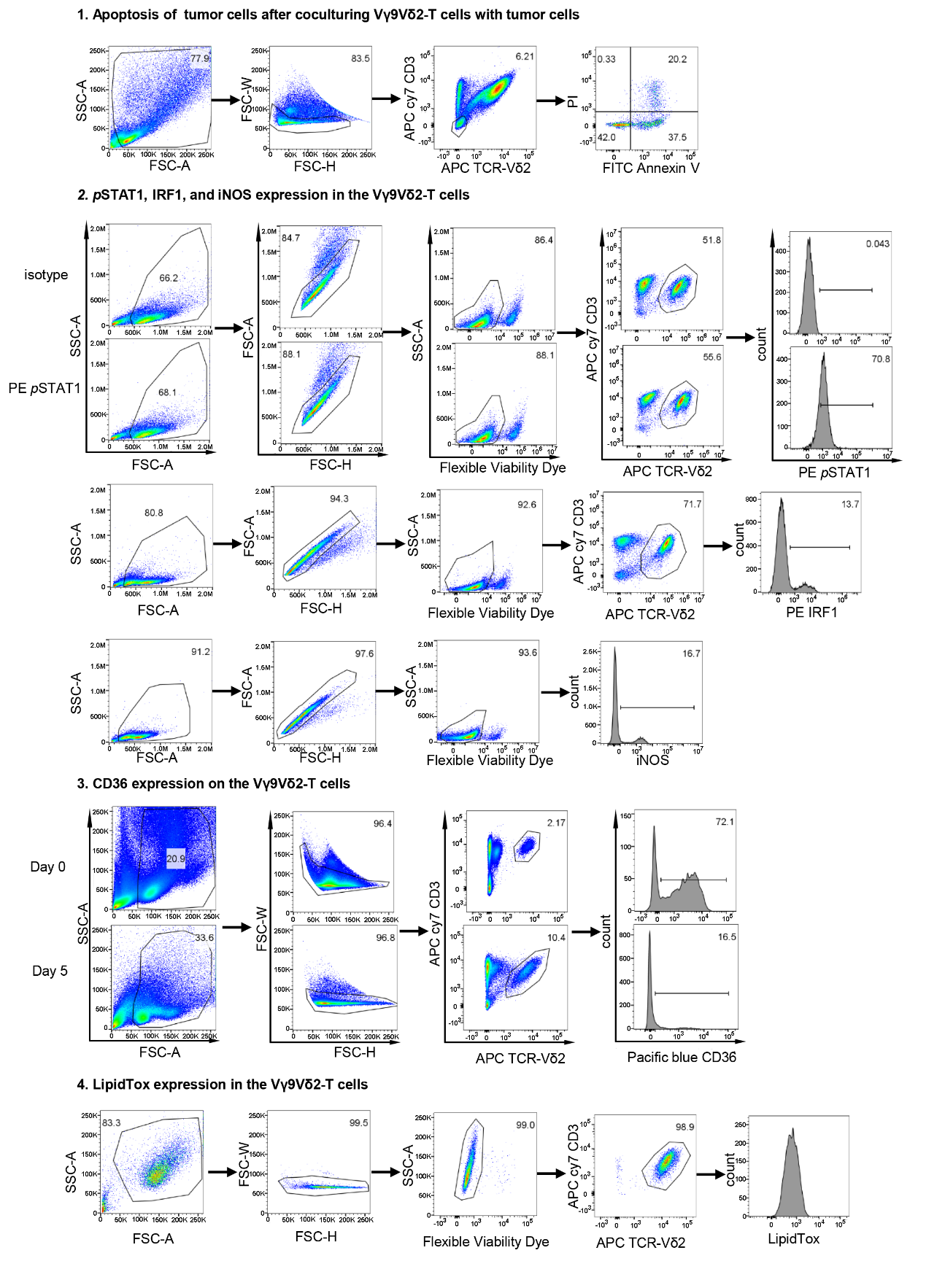


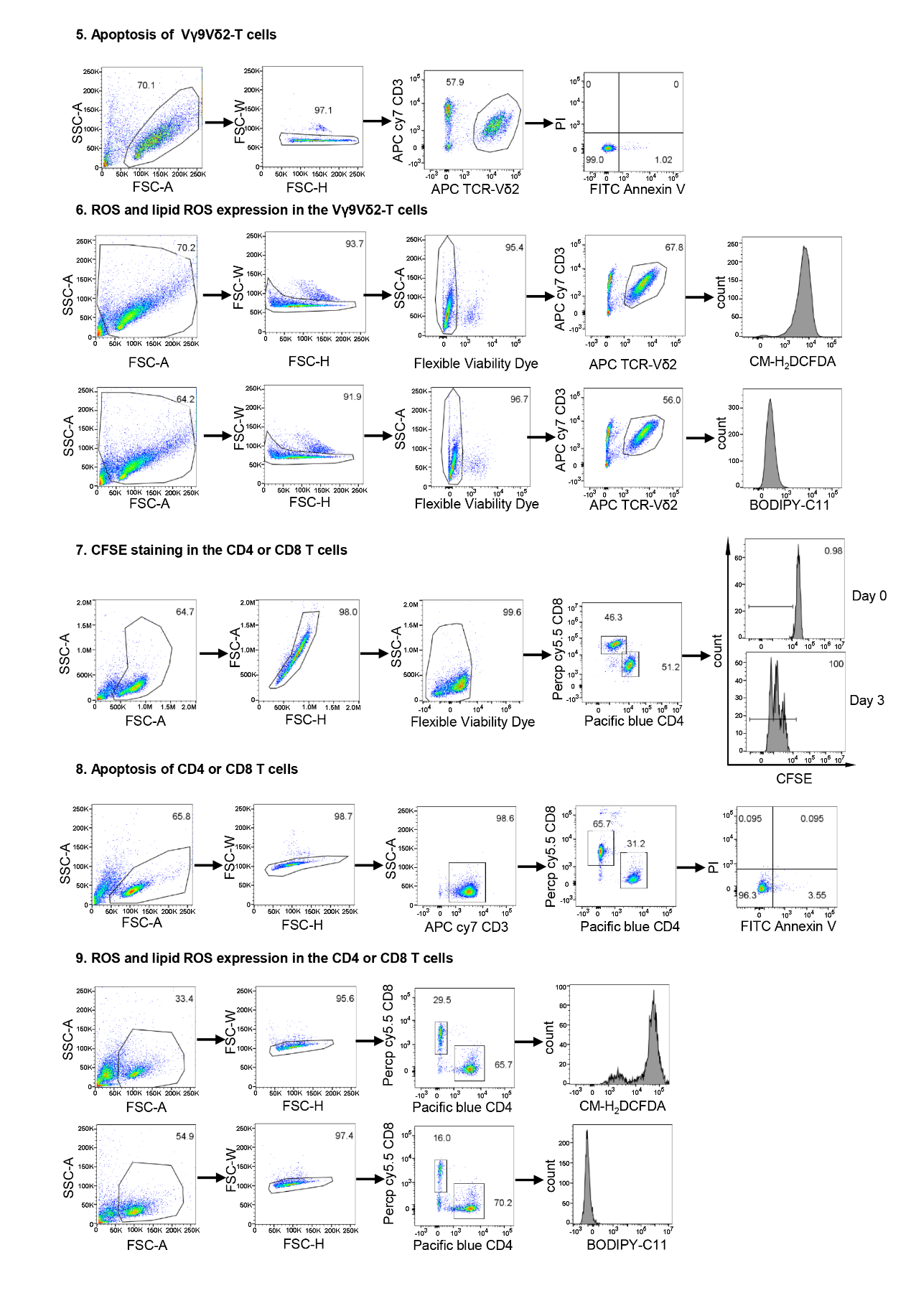

Supplement: Supplementary file 1 — supplemental materials [file 41392_2025_2295_MOESM1_ESM.docx]
